# Brain region-specific susceptibility of Lewy body pathology in synucleinopathies is governed by α-synuclein conformations

**DOI:** 10.1007/s00401-022-02406-7

**Published:** 2022-02-09

**Authors:** Laura de Boni, Aurelia Hays Watson, Ludovica Zaccagnini, Amber Wallis, Kristina Zhelcheska, Nora Kim, John Sanderson, Haiyang Jiang, Elodie Martin, Adam Cantlon, Matteo Rovere, Lei Liu, Marc Sylvester, Tammaryn Lashley, Ulf Dettmer, Zane Jaunmuktane, Tim Bartels

**Affiliations:** 1 Dementia Research Institute, University College London, London, UK; 2 Institute of Aerospace Medicine, German Aerospace Center, Cologne, Germany; 3 Department of Neuromuscular Diseases, UCL Queen Square Institute of Neurology, London, UK; 4 Department of Neurosurgery, NYU Grossman School of Medicine, New York, NY USA; 5 Department of Neurology, UW Medicine, Seattle, WA USA; 6 Ann Romney Center for Neurologic Diseases, Brigham and Women’s Hospital, Harvard Medical School, Boston, MA USA; 7 Department of Medical Sciences, University of Turin, Turin, Italy; 8 Medical Faculty, Core Facility Mass Spectrometry, Institute of Biochemistry and Molecular Biology, University of Bonn, Bonn, Germany; 9 Division of Neuropathology, National Hospital for Neurology and Neurosurgery, University College London NHS Foundation Trust, Queen Square, London, UK; 10 Queen Square Brain Bank for Neurological Disorders, UCL Queen Square Institute of Neurology, Queen Square, London, UK; 11 Department of Clinical and Movement Neurosciences, University College London, London, UK

**Keywords:** α-Synuclein, PD, DLB, Multimers, Aggregation, Aggregation transmission

## Abstract

The protein α-synuclein, a key player in Parkinson’s disease (PD) and other synucleinopathies, exists in different physiological conformations: cytosolic unfolded aggregation-prone monomers and helical aggregation-resistant multimers. It has been shown that familial PD-associated missense mutations within the α-synuclein gene destabilize the conformer equilibrium of physiologic α-synuclein in favor of unfolded monomers. Here, we characterized the relative levels of unfolded and helical forms of cytosolic α-synuclein in post-mortem human brain tissue and showed that the equilibrium of α-synuclein conformations is destabilized in sporadic PD and DLB patients. This disturbed equilibrium is decreased in a brain region-specific manner in patient samples pointing toward a possible “prion-like” propagation of the underlying pathology and forms distinct disease-specific patterns in the two different synucleinopathies. We are also able to show that a destabilization of multimers mechanistically leads to increased levels of insoluble, pathological α-synuclein, while pharmacological stabilization of multimers leads to a “prion-like” aggregation resistance. Together, our findings suggest that these disease-specific patterns of α-synuclein multimer destabilization in sporadic PD and DLB are caused by both regional neuronal vulnerability and “prion-like” aggregation transmission enabled by the destabilization of local endogenous α-synuclein protein.

**Supplementary Information:**

The online version contains supplementary material available at 10.1007/s00401-022-02406-7.

## Introduction

The protein α-synuclein (αS) is the major constituent of pathological neuronal inclusions both in Parkinson’s disease (PD) and dementia with Lewy bodies (DLB) [56]. Despite initially being characterized as natively unfolded, data collected in the last decade suggest that αS can exhibit different conformations under physiological conditions, which in turn help in governing aggregation propensity. The cytosolic unfolded, monomeric form of αS (αS^U^) is aggregation prone and can misfold into soluble, toxic oligomers, protofilaments, and amyloid fibrils, forms that are associated with pathology. The unfolded αS^U^ form, initially described in the literature as the only physiological species, exhibits spontaneous aggregation dependent on increased protein concentrations or long incubations into the pathological amyloid fibrillar form (αS^F^), a pathological hallmark of synucleinopathies [36, 43]. Conversely, the cytosolic helically folded, multimeric form (αS^H^) resists disease-associated changes [4, 59]. Our and several other laboratories have demonstrated that physiological αS^H^ (mainly tetramers and lower abundance related hexamers and octamers) exhibits enhanced aggregation resistance, plays a role in synaptic vesicle release and inversely correlates with various genetic PD risk factors [2, 4, 9, 12–15, 19, 21, 22, 26, 29, 30, 33, 38, 44–46, 49, 59, 61, 62]*.* Different techniques have been applied to prove the presence and analyze the structural identities, composition and aggregation properties of αS^H^ and αS^U^ in in vitro, ex vivo and in vivo models, e.g., non-denaturing Blue Native PAGE [4, 9, 22, 23, 59] and Clear Native PAGE [4], sedimentation equilibrium analytical ultracentrifugation [4, 14], gel filtration [9], YFP complementation [14], single particle electron microscopy [59], solution nuclear magnetic resonance [26, 59], high-resolution single-molecule force spectroscopy [44] or mass spectrometry [14, 22, 23, 59]. Moreover, αS^H^ stabilizing drugs [30, 45] have recently begun to enter clinical trials.

Accumulation of fibrillar αS^F^ in patients with PD with newly grafted neurons has been demonstrated in 2008/2009 [34, 35], implying that αS^F^ can spread from disease-affected to healthy tissue in a “prion-like” fashion. The templating “prion-like” properties of αS^F^ have been analyzed in various papers using different αS seeds (e.g., oligomers, recombinant αS, human brain homogenate, aggregated αS, preformed fibrils), target tissues and cells (e.g., CSF, olfactory mucosa, skin tissue, brain tissue, glial cells) in different sporadic and genetic synucleinopathies [3, 6, 7, 10, 16, 18, 24, 25, 28, 31, 37, 39, 40, 47, 48, 50, 52, 53, 55, 60].

However, whether this “prion-like” mechanism of a single protein can indeed lead to three distinct clinical diseases [PD, DLB, and multiple system atrophy (MSA)] is still unknown. Here, we show how local vulnerability of different brain regions, characterized by imbalances in the two physiological forms αS^U^ and αS^H^, contribute to the development of α-synucleinopathies. Our data presented here allow for the first time to explain region-specific vulnerability, patient stratification and how to potentially counteract “prion-like” aggregation transmission of αS through pharmacological stabilization of physiologically helical αS^H^.

## Materials and methods

### Brain samples

The experimental use of human brain samples was approved under the protocol number RA032611/1 by the University College London and the REC reference 18/LO/0721 (‘Queen Square Brain Bank for Neurological Disorders’).

### cDNA cloning

Single-mutation αS expression plasmids were generated from the pcDNA4 or pcDNA3/aS plasmid9 using the QuikChange II site-directed mutagenesis kit and appropriate primers.

### Cell lines and transfection

Cells were cultured at 37 °C in 5% CO_2_. Human BE(2)-M17 neuroblastoma cells (called M17D, ATCC number CRL-2267) were cultured in DMEM/F12 GlutaMAX (Thermo Fisher Scientific), 10% fetal bovine serum (Sigma) and 1% penicillin/streptomycin. Prior to the transfection, cells were seeded at a density of 1.5 × 106 cells/6 cm dish. HEK cells were cultured in DMEM with 10% fetal bovine serum (Thermo Fisher Scientific) and 1% penicillin/streptomycin and l-glutamine (2 mM, Gibco). Transfections with αS wild type and fPD mutants (A30P, E46K, G51D, A53T, H50Q) were carried out using Lipofectamine 2000 according to the manufacturer’s instructions. Cells were harvested 48 h after transfection, pelleted, snap frozen and further processed by cross-linking. HEK293 αS C-term Strep II-tagged lysates were purchased from GeneScript.

### Protein extraction

Frozen brain or cell pellet samples were manually homogenized in 1 × PBS/protease inhibitor/phosphatase inhibitor (Sigma, Thermo Fisher Scientific). Tissue suspensions were lysed by sonication (Sonic Dismembrator model 300, setting 40 for 15 s at 4 °C or Fisher Scientific Model 705 Sonic Dismembrator, sonication of amplitude 5% for 15 s at 4 °C). Samples were ultracentrifuged at 100,000*g* for 1 h at 4 °C. The resultant supernatant contained soluble proteins. To separate membrane proteins from insoluble proteins, the pellet from the homogenized brain tissue was resuspended in 1 ml OG-RIPA buffer (0.5% Nonidet P-40 substitute (NP-40, Pan Reac), 0.5% sodium deoxycholate (Sigma), 0.1% sodium dodecyl sulfate (SDS, Sigma), and 10 mM calcium chloride dihydrate (Sigma) with 2% *n*-octyl-*ß*-d-glucoside (abcam) and centrifuged at 175,000*g* for 30 min at 4 °C. The supernatant containing membrane-associated proteins was collected. The remaining pellet containing insoluble proteins was resuspended in 200 µl 8 M urea (Sigma)/5% SDS (Sigma)/PBS (Sigma), sonicated for 30 s (5 s on, 5 s off setting, amplitude 20%, room temperature (RT), Fisher Scientific Model 705 Sonic Dismembrator), and boiled for 10 min at 100 °C. Protein concentrations were measured with the Pierce BCA protein assay kit (Thermo Fisher Scientific) according to the manufacturer’s instructions.

### Purification of αS from erythrocytes

Freshly collected and washed erythrocytes were resuspended in threefold volume of ACK lysing buffer (Lonza, Walkersville MD, USA). (NH_4_)_2_SO_4_ to a final concentration of 25% was added and incubated at 4 °C for 30 min. The lysate was centrifuged (20,000*g*, 20 min), and the supernatant brought up to 50% (NH_4_)_2_SO_4_. The pellet was washed several times in 55% (NH_4_)_2_SO_4_ to remove excess hemoglobin. The sample was centrifuged at 20,000*g* for 20 min and the pellet resolubilized in 50-fold volume of 50 mM phosphate buffer, pH 7.0, 1 M (NH_4_)_2_SO_4_. Five millilitre of the resultant solution was injected onto a 5 ml HiTrap phenyl hydrophobic interaction column (GE Healthcare) equilibrated with 50 mM phosphate buffer, pH 7.0, 1 M (NH_4_)_2_SO_4_. αS was eluted with a 1–0 M (NH_4_)_2_SO_4_ gradient in 50 mM phosphate buffer, pH 7.0 (αS eluted at ~ 0.75 M (NH_4_)_2_SO_4_).

### Cross-linking of lysate

Twenty microgram of total protein in a total volume of 25 µl was incubated with 10 µl 5 mM DSG (Thermo Fisher Scientific, final concentration 1.43 mM, DSG was first dissolved in 50 µl of anhydrous DMSO and further in PBS pH 7.4 to a final volume of 1 ml) for 30 min at 37 °C shaking or rotating. The reaction was quenched with 3.5 µl 1 M Tris–HCl, pH 7.6 (Sigma), for 5 min shaking at RT. Samples were analyzed in biological duplicates and technical triplicates. The cross-linker disuccinimidyl suberate (DSS, Thermo Scientific Pierce) was used for validation.

### Immunoblotting (Western blot, WB)

Samples were boiled at 90 °C in 10 µl 4 × NuPage LDS sample buffer (Novex)/1:10 *β*-mercaptoethanol (Sigma) for 5 min. Samples were electrophoresed at maximum 200 V on NuPAGE Bis Tris Midi gels (Invitrogen) with NuPage MES-SDS running buffer (Invitrogen) and the SeeBlue Plus2 pre-stained molecular weight marker (Invitrogen). The total volume of each sample containing 20 µg total protein was loaded on each lane. After electrophoresis, gels were incubated in 20% ethanol (Decon Laboratories) for 5 min at RT and electroblotted onto iBlot 2 NC Regular Stacks (Invitrogen) using the iBlot Dry Blotting preset 7 min blotting program. The membrane was briefly rinsed in ultrapure water and incubated in 4% paraformaldehyde/PBS (Alfa Aesar) for 30 min at RT. Some membranes were stained with 0.1% Ponceau (Biotium) and rinsed with ultrapure water. Membranes were blocked in Odyssey blocking buffer (PBS)/PBS buffer 1:1 (LI-COR) or casein buffer 0.5% (BioRad) for 30 min at RT. After blocking, membranes were incubated with primary antibodies overnight at 4 °C. Membranes were briefly rinsed in PBS-Tween 0.1% and then washed 3 × 10 min in PBS-Tween 0.1%. Membranes were incubated with the corresponding secondary LI-COR antibodies (1:20,000 in Odyssey blocking buffer (PBS)/PBS 1:1/Tween 0.1% at RT for 1 h in the dark). Membranes were briefly rinsed in PBS-Tween 0.1% and then washed 3 × 10 min in PBS-Tween 0.1% in the dark. Membranes were imaged on a LI-COR Odyssey CLx imaging system (settings: custom, western, quality low, resolution 169 µM, focus offset 0.0, auto intensity).

### Antibodies

Antibodies used were 2F12 to αS[12] (MABN1817, Merck, WB 1:5000), SOY1 to αS (MABN1818, Merck), and anti-DJ-1 [5] (or GeneTex, WB 1:2000). Anti-Tau (EP2456Y, abcam, WB 1:5000), anti-β tubulin III (T2200, Sigma-Aldrich, WB 1:5000), anti-heat shock protein 70 (HSP70, ab181606, abcam, WB 1:1000), anti-14-3-3 beta (ab15260, abcam, WB 1:500), anti-β actin (ab8227, Abcam, WB 1:5000), and anti-HSP90 (HEK: ab clone 16F1, ADI-SPA-83, Enzo, WB 1:1000, huBrain: ab PA3-013, Invitrogen, WB 1:500).

### αS-specific ELISA

Multi-array 96 HB plates (MSD) were coated with the capture antibody anti-αS 2F12 (200 ng 2F12[12] in PBS, 30 µl/well) and incubated over night at 4 °C. The next day, the remaining liquid was removed, and the plates were blocked with 5% Blocker A (MSD) in PBS-Tween 0.1% (150 µl/well, Sigma) shaking for 2 h at RT. Plates were washed 5× with PBS-Tween 0.1% (150 µl/well). Standards (recombinant αS, highest concentration 1 ng, ratio for serial dilution 1:4, 30 µl/well) and protein (30 µl/well, soluble protein fraction 1:500, membrane (OG-RIPA) and insoluble (UREA/SDS fraction 1:100) were diluted in 1% Blocker A in PBS-Tween 0.1% and the plates were incubated shaking for 2 h at RT. The remaining liquid was removed and the plates were washed 5× with PBS-Tween 0.1% (150 µl/well). The sulfo-tagged detection antibody SOY1 (50 ng SOY1 in 1% Blocker A (MSD)/PBS-Tween 0.1%, 30 µl/well) was added and the plate incubated shaking for 1 h at RT in the dark. The remaining liquid was removed, and the plates were washed 5× with PBS-Tween 0.1% (150 µl/well). 2× read buffer (MSD)/MilliQ water was added (150 µl/well) and the plates were immediately measured using an MSD Sector 2400 imager or MSD Model No. 1300, QuickPlex SQ 120 according to the manufacturer’s instructions. All samples and standards were analyzed in technical duplicates.

### Immunoprecipitation of αS

αS from brain tissue was immunoprecipitated using the Pierce Direct IP Kit (Thermo Fisher Scientific) according to the manufacturer’s instructions. The 2F12 to αS[12] was used as a capture antibody (300 µg/reaction). At least, 800 µg total protein was used for each cross-linking of lysate reaction using 5 mM DSG (final concentration in total volume 1.43 mM). Volumes of wash and incubation buffers were adapted to the total volume input of the sample. Elution fractions were further concentrated using Amicon concentration columns (Millipore) according to the manufacturer’s instructions. The fractions were checked for the presence of αS monomer and multimer using immunoblotting and Coomassie staining. Negative controls included the incubation and clearance of samples using the Pierce Control Agarose Resin (non-amine reactive) and the incubation of samples on the AminoLink Plus™ Coupling Resin column without any antibody. Normal mouse IgG was used as a positive control (200 µg/AminoLink Plus™ Coupling Resin column, sc-2025, Santa Cruz Biotechnology). αS from HEK293 αS C-term Strep II-tagged lysates was immunoprecipitated using StrepTrap 5 ml columns. Briefly, cells were lysed, ultracentrifuged (100,000*g* at 4 °C for 1 h) and the protein content measured in the supernatant. Cross-linking was performed according to the protocol above and the cross-linked lysate was loaded onto equilibrated Strep Tag 5 ml columns. Unbound samples were washed out with PBS buffer, pH 7.4, and the sample eluted with elution buffer (2.5 mM desthiobiotin in PBS, pH 7.4). For mock controls, HEK without αS tag were subjected to immunoprecipitation.

### Size exclusion chromatography (SEC)

Immunoprecipitated αS fractions were injected on a Superdex 200 (10/300 GL) column (GE Healthcare) at room temperature and eluted with 50 mM NH4Ac (pH 7.4) while measuring (in-line) the conductivity and the 280-nm absorption of the eluate at 0.7 ml/min. For size estimation, a gel filtration standard (Bio-Rad) was run on the column, and the calibration curve was obtained by semilogarithmic plotting of molecular weight versus the elution volume divided by the void volume. Immunoblotting was performed to identify the fractions containing αS multimers and monomers.

### Circular dichroism spectroscopy (CDS)

After SEC separation, samples containing αS multimers or monomers were exchanged into 10 mM ammonium acetate using Zeba spin desalting columns (Thermo Fisher), lyophilized, and resuspended in 10 mM ammonium acetate at a concentration of approximately 10 μM. αS samples were added to a 1 mM path length quartz cuvette for far-UV CD and analyzed using a J-1500 CD spectrometer (Jasco) at 25 °C. Temperature control with an accuracy of 0.1 °C was achieved with a heating/cooling accessory equipped with a Peltier element (PFD-425S) connected to a water thermostatic bath. Buffer spectra were recorded and subtracted.

### Multi-angled light scattering (MALS)

Immunoprecipitated and SEC-separated αS multimers and monomers were loaded at a flow rate of 0.15 ml/min onto a Superdex 200 3.2/300 GL Increase column (GE Healthcare) previously equilibrated in 50 mM ammonium acetate, pH 7.4. The column was connected in line to a Dawn 8 + MALS detector (Wyatt Technology) using a laser emitting at 690 nM and by refractive index measurement using an Optilab T-rex (Wyatt Technology Corp.).

### PFF generation

Five milligram per millilitre recombinant αS in PBS was aggregated for 3 days at 37 °C with nutation to form thioflavin T-positive fibrils. To generate soluble PFFs, αS fibrils were diluted to 1 mg/ml and sonicated at power level 50 for 3 × 10 s using a Sonic Dismembrator model 300 (Fisher). Aliquots of the resultant material were flash-frozen in liquid nitrogen and stored at − 80 °C.

### Preformed fibril (PFF) transfection

All reagents were purchased from Thermo Fisher Scientific unless otherwise noted. Transfected M17D were incubated with 0.5 µg/ml αS PFF. After 48 h, the medium was aspirated, and cells were harvested by scraping and washed 2× in cold PBS (500*g* spin for 5 min). Cell pellets were resuspended in 1× PBS/protease inhibitor and sonicated with a Sonic Dismembrator model 300 (microtip setting 40; 2 × 15 s). Cells were spun at 20,000*g* for 10 min. The supernatant was kept (“cytosol”) and the pellet was incubated in 1% Triton X-100 at 4 °C for 30 min under nutation. After a 20,000*g* spin for 10 min, the supernatant was kept (“membrane fraction”) and the pellet dissolved in 5% SDS at 100 °C for 10 min to give the “insoluble αS” fraction. Relative αS content in each fraction was analyzed by SDS-PAGE/Western blot and ELISA. For the SCDi experiments, M17D/αS-WT::YFP[30] cells were monitored on an IncuCyte Zoom machine (Essen Bioscience). Cells were seeded at a density of 0.1*10°6 cells/ml in a 12-well plate. Twenty-four hours after seeding, a fraction of the cells was treated with 10 µM SCDi or DMSO. Twenty-four hours after the treatment, some cells were seeded with 0.5 µg/ml αS^F^. Another 48 h and 96 h after seeding, SCDi or DMSO was added again. The experiments were carried out in biological duplicates. The IncuCyte settings and analysis were performed analogous to Imberdis et al. [30]. For the seeding experiment, M17D human neuroblastoma cells constitutively expressing wt αS were analyzed in biological and technical duplicates, and cross-linking was performed analogous to brain samples. Cells were seeded at a density of 0.1*10°6 cells/ml in a 12-well plate. Twenty-four hours after seeding, some cells were seeded with 0.5 µg/ml αS^F^.

### Thioflavin T (ThT) binding

To detect amyloid fibril growth, a discontinuous assay was used. Aliquots (10 μl) were removed from each purified αS sample (lyophilized from 50 mM ammonium acetate, pH 7.4, and agitated at 37 °C at a concentration of 75 μM in 20 mM Bis–Tris propane, 100 mM LiCl, pH 7.4) and added to 2 ml of a 10 μM thioflavin T (ThT) solution in 10 mM glycine buffer, pH 9. Fluorescence was directly quantified on a Varian Eclipse fluorescence spectrophotometer at 20 °C by exciting at 444 nM and scanning the emission wavelengths from 460 to 550 nM with slit widths set at 5 nM (PMT at 750 V).

### Mass spectrometry

HEK293 αS C-term Strep II-tagged lysates were cross-linked and immunoprecipitated as described above and the αS^H^ and αS^U^ were separated via SEC and run on an SDS-gel. Gel pieces containing the bands of interest were cut out and lyophilized prior to mass spectrometry analysis. Gel slices were subjected to gel digestion. In brief, slices were washed consecutively with water, 50% acetonitrile (ACN), and 100% ACN. Proteins were reduced with 20 mM DTT in 50 mM ammonium bicarbonate and alkylated with 40 mM acrylamide (in 50 mM bicarbonate). The slices were washed again and dehydrated with ACN. Proteolysis was performed with 330 ng chymotrypsin or trypsin (sequencing grade Promega, Mannheim, Germany) at 37 °C overnight. The peptide extracts were dried in a vacuum concentrator and stored at − 20 °C. Dried peptides were dissolved in 10 µl 0.1% formic acid (solvent A). Two microlitres was injected onto a C18 analytical column (self-packed 300 mM length, 100 µM inner diameter, ReproSil-Pur 120 C18-AQ, 3 µM, Dr. Maisch GmbH, Ammerbuch-Entringen, Germany). Peptides were separated during a linear gradient from 2 to 35% solvent B (90% acetonitrile, 0.1% formic acid) within 90 min at a flow rate of 300 nl/min. The nanoHPLC was coupled online to an Orbitrap Lumos mass spectrometer (Thermo Fisher Scientific, Bremen, Germany). Peptide ions between 330 and 1600 m/z were scanned in the Orbitrap detector with a resolution of 60,000 (maximum injection time 50 ms, AGC target 400,000). Precursor ions (threshold 25,000) were subjected to higher energy collision-induced dissociation within a 2.5 s cycle and fragments were analyzed in the Orbitrap detector (maximum injection time 22 ms, resolution = 15,000). Fragmented peptide ions were excluded from repeat analysis for 15 s.

### Semiquantitative Lewy body score

Lewy body pathology density was assessed semiquantitatively on formalin-fixed paraffin-embedded tissues at 20× magnification (Leica DM3000 microscope), using revised McKeith staging scheme of αS pathology [42], with the stages ranging from 0.5 to 4. Stage 0.5 was assigned if a single Lewy body was seen in a region of interest after screening several nearby fields. Stage 1 was assigned if a single Lewy body was consistently seen per each field after screening several nearby fields, and Stage 1.5 if a single Lewy body was seen per field with rare additional two Lewy bodies per single nearby field. Stage 2 was assigned if two to three Lewy bodies were seen per field, and Stage 2.5, if twio to three Lewy bodies were seen per field and rare additional four Lewy bodies per nearby field. Stage 3 was assigned if 4–10 Lewy bodies were consistently seen per field and Stage 3.5, if 11–20 Lewy bodies were seen per field. Stage 4 was assigned if more than 20 Lewy bodies were seen per field. The following anatomical regions were assessed: amygdala, transentorhinal cortex, peri- and parastriate cortex, and cortical regions of the insula, anterior cingulate gyrus, middle temporal gyrus, Heschl’s gyrus, anterior middle frontal gyrus, and inferior parietal lobule.

### Protein ratio determination and statistics

Western Blot: the ratio of protein multimers to monomers was analyzed using the Image Studio software western analysis according to the manufacturer’s instructions (background subtraction: median, border with 3, top/bottom). Data analysis (except for correlation and regression analysis) was performed using GraphPad Prism 7 (GraphPad Software, La Jolla, CA, USA). Statistical significance was determined by Mann–Whitney test (*p* < 0.05, two-tailed). Samples are displayed as mean ± SD. Linear regression analysis was performed using R. Additional R software packages included “devtools” (https://github.com/r-lib/devtools), “easyGgplot2” (https://github.com/kassambara/easyGgplot2) and “Hmisc” (https://github.com/harrelfe/Hmisc). Samples were coded as follows. Gender: male = 1, female = 2. McKeith: neocortical = 3, limbic = 2, brainstem = 1, controls (no McKeith staging) were set to 0. CERAD: none = 0, sparse = 1, mild = 2, moderate = 3, frequent = 4. Braak LB staging: controls were set to 0, PD and DLB patients were coded according to evaluated Braak LB staging. ELISA: raw data from the MSD Discovery Workbench was imported into Excel 2010 (Microsoft) for background calculation and subtraction. Values from Excel were imported into GraphPad Prism 7 (GraphPad Software, La Jolla, CA, USA) for interpolation together with the known standard concentrations (standard curve: sigmoidal, 4PL, X is log (concentration, no special handling for outliers). The final concentration of each analyzed sample was calculated in Excel according to the dilution factor. Mass spectrometry: raw data processing and analysis of database searches were performed with Proteome Discoverer software 2.4.0.305 (Thermo Fisher Scientific). The protein-specific peptide identification was done with an in-house Mascot server version 2.6.1 (Matrix Science Ltd, London, UK) from Proteome Discoverer. MS2 data were searched against a database of common contaminants (cRAP of the Global Proteome Machine), and human SwissProt sequences. Precursor ion m/z tolerance was 10 ppm, and fragment ion tolerance 0.02 Da. Tryptic peptides with up to two missed cleavages were searched, propionamide was set as a static modification of cysteines, and oxidation as a dynamic modification of methionine. Mascot results from searches against SwissProt were sent to the Percolator algorithm_ENREF_3 version 3.02 as implemented in Proteome Discoverer 2.4. If the data did not permit use of Percolator, PSMs were evaluated by a target-decoy procedure. Spectra without high confidence (FDR 1%) matches were sent to a second round Mascot search with semi-specific enzyme cleavage. The false discovery rate of proteins in these samples was 0.6%.

## Results

### α-Synuclein exists in human brain in an equilibrium between helically folded homo-multimeric and unfolded monomeric species

**Fig. 1:**
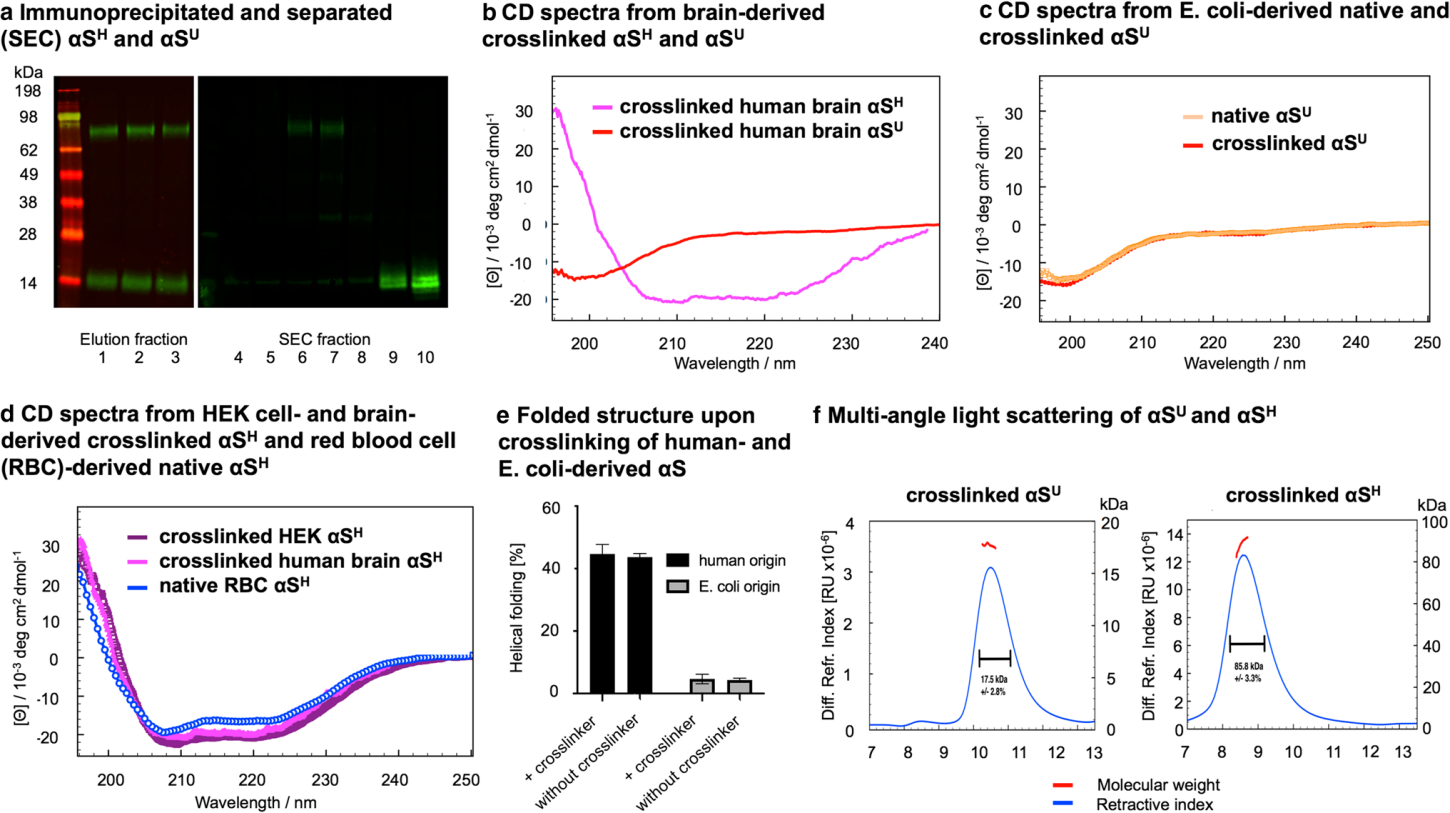
Cytosolic, multimeric brain-derived αS is helically folded and exhibits a mass of 86 kDa as demonstrated by circular dichroism spectroscopy (CDS) and multi-angle light scattering (MALS) analyses of purified αS^H^ and αS^U^ a Elution fractions 1–3 of a 2F12 anti-αS column incubated with a cross-linked control brain lysate showing the purification of the αS protein. On the right side, fractions 4–10 after size exclusion chromatography (SEC) are displayed showing the separation of αS^H^ and αS^U^ for downstream CDS and MALS analysis. b CDS of immunoprecipitated and separated (SEC) αS^H^ and αS^U^ from human frontal cortex control brain tissue upon cross-linking of brain lysate. The αS^H^ from human brain exhibits an α-helical secondary structure of ~ 50%. c CDS of recombinant αS from *E. coli*. The recombinant αS is unfolded (~ 5% helical fold, ~ 95% unfolded, monomeric). The unfolded structure of recombinant αS from *E. coli* remains after cross-linking (~ 5% helical fold, ~ 95% unfolded, monomeric). d CDS of purified, native αS^H^ from red blood cells (RBC) exhibits a secondary structure of ~ 45% helical fold and ~ 55% unfolded[4]. Cross-linked αS^H^ from HEK cells exhibits an α-helical secondary structure of ~ 48% helical fold and 52% unfolded. The cross-linked αS^H^ from human brain exhibits an α-helical secondary structure of ~ 50%. e Folded structure of αS without and with cross-linking demonstrating the difference from human and recombinant origin. f Immunoprecipitated αS^U^ from frontal cortex tissue of a control was separated from αS^H^ by SEC and analyzed via MALS demonstrating a mass of ~ 18 kDa. Immunoprecipitated αS^H^ from frontal cortex tissue of a control was separated from αS^U^ by SEC and analyzed via MALS demonstrating a mass of ~ 86 kDa

To address the question of whether the equilibrium of αS^U^ and αS^H^ also plays an important role in sporadic synucleinopathies, we adapted our established [4, 12, 29] multimer assay (using cross-linking and Western blot analysis of lysate) for frozen human post-mortem tissue (Online Resource Figs. 1, 2, 3, 4, 5). This protocol revealed a prominent cytosolic ~ 80 kDa and 14 kDa αS species (Fig. 1a; Online Resource Figs. 2, 3, 4, 5) as described previously [12, 29, 38]. The two cytosolic αS species corresponded to a 86 kDa helically folded homo-multimer and a 14 kDa monomer, respectively, as confirmed by circular dichroism (CD) spectroscopy, Multi-angle light scattering and Western blotting were conducted on purified 14 kDa and 86 kDa species from human brain tissue and HEK cells (Fig. 1a, b, f; Online Resource Figs. 6, 7). Mass spectrometry and Western Blot analysis of the 86 kDa species showed only the presence of αS compared to controls, indicating its homo-multimeric nature (Online Resource Table 1, Online Resource Figs. 8, 9, 10, 11). We confirmed that the cross-linking procedure did not lead to an artificial production of αS^H^ using recombinant *E. coli*-derived protein in contrast to brain tissue (which led only to cross-linked αS^U^), HEK cells or red blood cells (yielding cross-linked αS^H^) and non-denaturing purification from both human tissue and bacterial cells (leading to the same species obtained under cross-linking conditions, Fig. 1c–e). Figure 1 shows the biophysical analysis of physiological αS found in the cytosol. Insoluble or amyloid-type aggregated forms of pathological αS are not found in the cytosol (they represent typically only ~ 0.5% of total protein in this subcellular compartment) as we have shown before in human brain [54]) and are therefore not displayed.

### The equilibriums of cytosolic helically folded and unfolded physiological αS is disturbed in PD and DLB patients

**Fig. 2:**
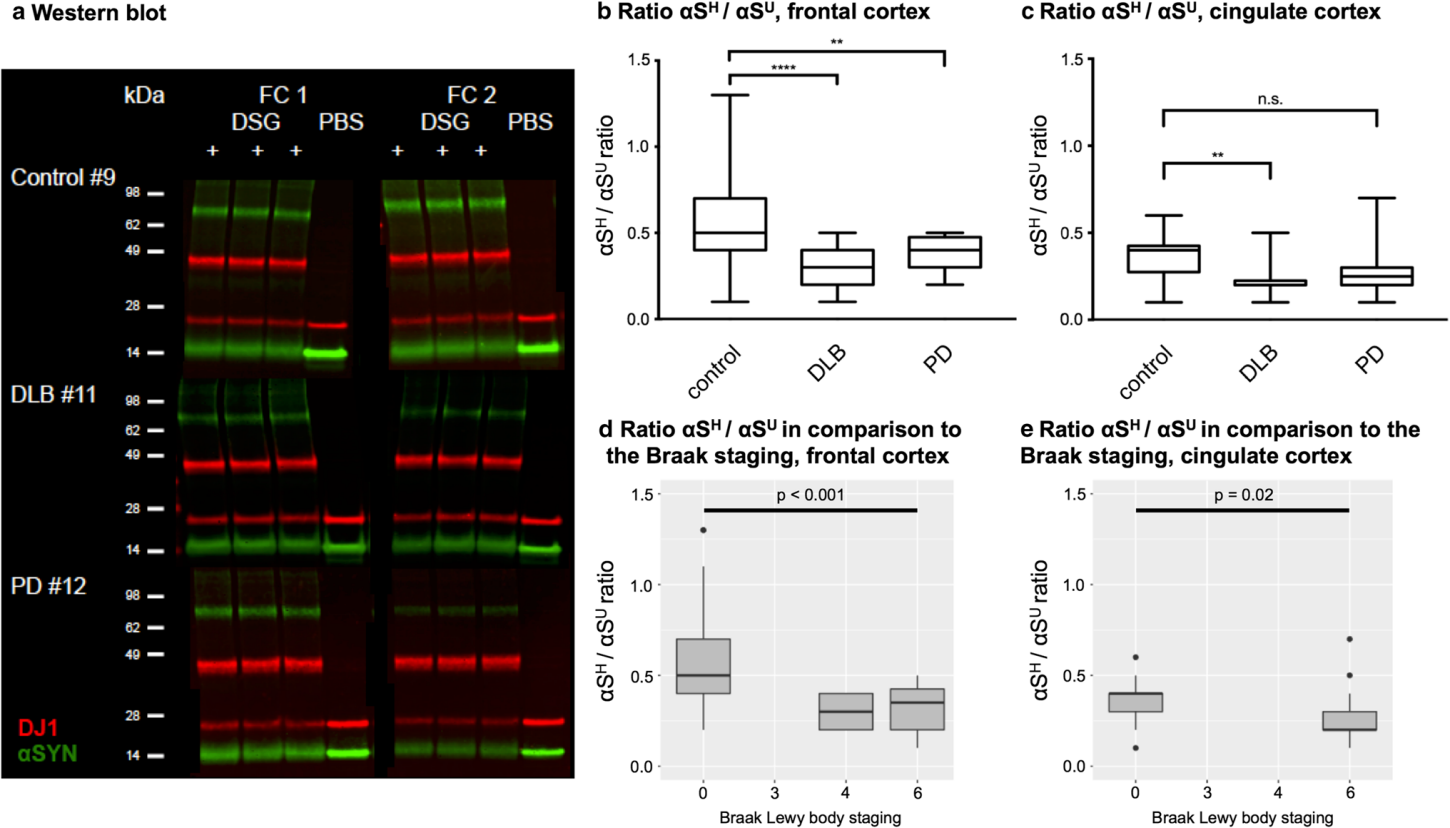
Equilibriums of cytosolic helically folded and unfolded physiological αS is disturbed in PD and DLB patients. **a** Representative Western blot of αS^H^ and αS^U^ in controls, DLB and PD patients. Each piece of brain was analyzed in duplicates (frontal cortex FC1 and FC2). The cross-linking reaction was performed in technical triplicates alongside one non-cross-linked (PBS) sample. The Western blot demonstrates reduced αS^H^ to αS^U^ ratios in DLB and PD patients compared to the control. DSG “+” = cross-linked sample. DSG “−” = non-cross-linked sample. Green = αS, red = DJ1. **b** Significant reduction of αS^H^/αS^U^ ratio in the frontal cortex comparing controls (*n* = 19) with DLB (*n* = 14) and PD (*n* = 8) patients. **c** Significant reduction of αS^H^/αS^U^ ratio in the cingulate cortex comparing controls (*n* = 7) to DLB (*n* = 7) patients. No significant alteration of the αS^H^/αS^U^ equilibriums in the cingulate cortex comparing controls (*n* = 7) and PD (*n* = 6) patients. **d** Significant difference of decreased αS^H^/αS^U^ ratios and increased Braak LB staging of the frontal cortex (*p* < 0.001). **e** Significant difference of decreased αS^H^/αS^U^ ratios and increased Braak LB staging of the cingulate cortex (*p* = 0.02)

Next, we assessed the ratio of cytosolic helical and unfolded αS (αS^H^/αS^U^) in four different brain regions (entorhinal cortex, cingulate cortex, frontal cortex and striatum) of 28 age-matched individuals, classified as neurological controls, 15 DLB patients and 15 sporadic PD patients (Table 1). Representative pictures of Western blot analyses from controls and DLB and PD patients are displayed in Fig. 2a and Online Resource Fig. 12.

**Table 1:** Overview of samples and processed brain tissue for Fig. 2

Case #	Age at death [years]	Gender	Disease duration [years]	PMI [hours]	McKeith	Braak LB	Braak NFT	CERAD	FC	CC	EC	Str.
Control 1	77	Male		83			2	Absent		x		
Control 2	74	Female		53			3	Absent		x		
Control 3	95	Female		39			4	Moderate	x			x
Control 4	99	Female		32			4	Frequent	x			x
Control 5	87	Male		39			1	Absent	x			x
Control 6	57	Female		13					x			
Control 7	86	Male		10					x			
Control 8	92	Male		24			2	Absent	x			x
Control 9	92	Male		23			2	Sparse	x			x
Control 10	54	Male		6			0	Absent	x			x
Control 11	60	Male							x			
Control 12	75	Female					4		x			
Control 13	81	Female					2		x			
Control 14	75	Female					3		x			
Control 15	81	Male					3		x			
Control 16	78	Male					2		x			
Control 17	79	Female					2		x			
Control 18	70	Male					2		x			
Control 19	63	Male					3		x			
Control 20	87	Female					3		x			
Control 21	88	Female					5		x			
Control 22	90	Male		46			4	Moderate			x	
Control 23	76	Male		79			2	Absent		x	x	
Control 24	84	Female		54			2	Absent		x	x	
Control 25	83	Female		99			2	Sparse		x	x	
Control 26	89	Male		156			3	Sparse		x	x	
Control 27	82	Female		91			2	Sparse		x	x	
Control 28	84	Male		79			1				x	
DLB 1	77	Male	2.5	29	Neocortical	6	2	Moderate		x		
DLB 2	71	Male	5	22	Neocortical	6	3	Moderate	x	x	x	
DLB 3	72	Male	8	89	Neocortical	6	3	Absent	x	x	x	
DLB 4	84	Male	10	72	Neocortical	6	2	Absent	x	x	x	
DLB 5	73	Female	6	73	Neocortical	6	3	Absent	x	x	x	
DLB 6	76	Male	8	13	Neocortical	6	2	Sparse	x			
DLB 7	81	Male	5	26	Neocortical	6	3	Moderate	x	x	x	
DLB 8	81	Male	3	81	Neocortical	6	3	Moderate	x	x	x	
DLB 9	60	Male	8	24	Neocortical	6	1	Moderate	x			x
DLB 10	67	Male	7	40	Neocortical	6	3	Moderate	x			x
DLB 11	79	Female		12	Neocortical	6	4	Sparse	x			
DLB 12	83	Male	8	54					x			x
DLB 13	63	Female	10				4		x			x
DLB 14	81	Female			Neocortical	6	3		x			
DLB 15	67	Female			Neocortical	6	4		x			
PD 1	42	Male	4	42	Neocortical	6	4			x	x	
PD 2	78	Male	3	24	Neocortical	6	0	Absent		x	x	
PD 3	83	Female	8	99	Neocortical	6	3			x	x	
PD 4	76	Male	6	85	Neocortical	6	6			x	x	
PD 5	76	Male	7	49	Neocortical	6	1	Sparse		x	x	
PD 6	82	Male	11	7	Neocortical	6	2	Absent		x	x	x
PD 7	81	Male	13	46	Neocortical	6	2	Absent	x			x
PD 8	74	Male	16	55	Neocortical	6	2	Absent	x			
PD 9	79	Female	15	65	Neocortical	6	2	Mild	x			x
PD 10	79	Female	14	27	Neocortical	6	4	Absent	x			x
PD 11	75	Male	22	11	Neocortical	6	2	Absent	x			x
PD 12	76	Male	10	29	Neocortical	6	2	Absent	x			
PD 13	87	Male		38	Limbic	3	1	Sparse				x
PD 14	73	Male		24	Limbic	4			x			
PD 15	87	Female		6	Limbic	4	2	Sparse	x			

*DLB* dementia with Lewy bodies, *PD* Parkinson’s disease, *PMI* post-mortem interval, *LB* Lewy bodies, *NFT* neurofibrillary tangles, *CERAD* Consortium to Establish a Registry for Alzheimer’s Disease, *FC* frontal cortex, *CC* cingulate cortex, *EC* entorhinal cortex, *Str.* striatum

We found that DLB patients exhibited a significant reduction of αS^H^/αS^U^ in the frontal cortex (*p* < 0.001, Fig. 2b) and cingulate cortex (*p* = 0.004, Fig. 2c). PD patients exhibited a significant decrease of αS^H^/αS^U^ in the frontal cortex (*p* = 0.001, Fig. 2b). We did not observe significant changes between patients and controls in the entorhinal cortex, a region typically affected by Lewy body (LB) pathology earlier in the disease course than neocortical regions (Online Resource Fig. 13a), the striatum which served as a control region (due to low LB burden, Online Resource Fig. 13b) or the internal control protein DJ1 (Online Resource Fig. 13c–f). Analysis of variance with regard to clinical and neuropathological features (summary Table 1) revealed a significant difference between the reduction of the αS^H^/αS^U^ and increased αS Braak LB staging in the frontal cortex (*p* < 0.001) and cingulate cortex (*p* = 0.02, Fig. 2d, e). A similar difference was observed between increased McKeith staging and reduced αS^H^/αS^U^ ratios (frontal cortex *p* < 0.001; cingulate cortex *p* = 0.02, Online Resource Fig. 14). A significant difference was also detected between decreased αS^H^/αS^U^ ratios and increased Braak neurofibrillary tangle (NFT) staging in the entorhinal cortex (*p* = 0.04, Online Resource Fig. 14). All other clinical features such as gender, age or post-mortem interval (Table 1) were not associated with the reduction of αS^H^/αS^U^ (data not shown). Hence, these data indicate a region-specific destabilization of physiological αS in brain regions affected by LB pathology.

### αS^H^ exhibits “prion-like” aggregation-resistant properties

**Fig. 3:**
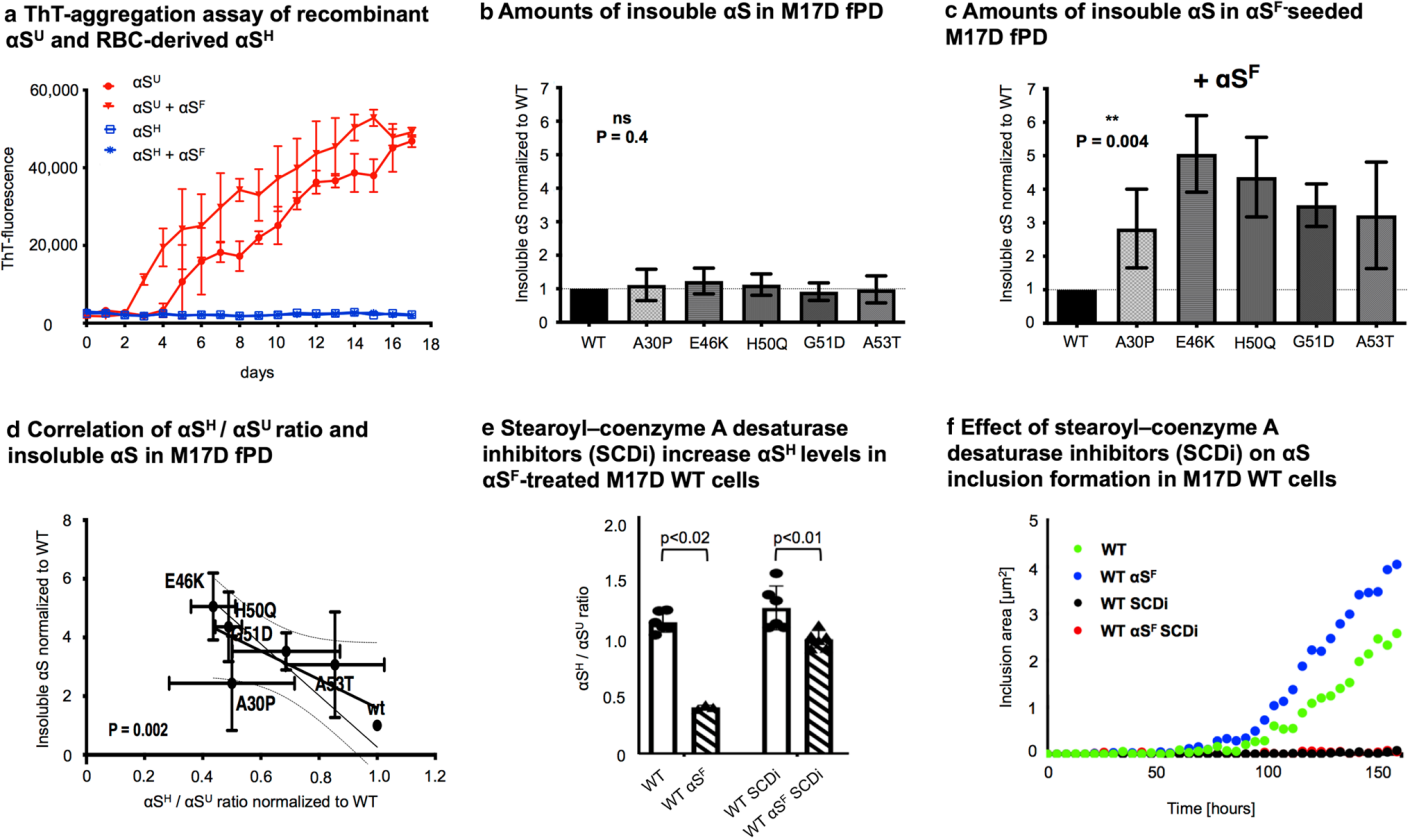
αS^H^ is resistant to spontaneous or “prion-like” induced aggregation compared to αS^U^ and modulates “prion-like” aggregation transmission in cellular models of disease. **a** Thioflavin T-fluorescence assay monitoring the aggregation of recombinant αS^U^ and purified αS^H^ from erythrocytes. In samples that were seeded, 10 nM recombinant fibrillar αS (αS^F^) (i.e., 1:1000) was added. The αS^H^ multimer demonstrated resistance to both spontaneous and seeded aggregation. **b** M17D cells transfected with wt αS, or the fPD related mutations A30P, E46K, H50Q, G51D and A53T. Cells with fPD mutations display equal amounts of insoluble αS. Cells were analyzed in biological triplicates. **c** αS^F^-seeded M17D cells display increased levels of insoluble αS. Cells were analyzed in biological triplicates. **d** αS^H^ destabilization correlates significantly (Deeming regression analysis) with susceptibility towards αS aggregation as measured by insoluble αS in M17D after 2 days of seeding (*p* = 0.002). Cells were analyzed in biological triplicates. **e** SCDi treated and αS^F^-seeded M17D cells exhibit increased αS^H^/αS^U^ ratios compared to αS^F^-seeded M17D wt cells. Cells were seeded at a density of 0.1*10°6 cells/ml in a 12 well plate Twenty-four hours after seeding, a third fraction of the cells were treated with 10 µM SCDi or DMSO. Twenty-four hours after the treatment, some cells were seeded with 0.5 µg/ml αS^F^. Another 48 h and 96 h after seeding, SCDi or DMSO was added again. The cross-linking experiments were carried out in 6 independent experiments. **f** SCDi prevent the formation of pathological αS inclusions even in the presence of αS^F^. Cells were seeded at a density of 0.1*10°6 cells/ml in a 12 well plate. Twenty-four hours after seeding, a third fraction of the cells were treated with 10 µM SCDi or DMSO. Twenty-four hours after the treatment, some cells were seeded with 0.5 µg/ml αS^F^. Another 48 h and 96 h after seeding, SCDi or DMSO was added again. Aggregation was monitored until a decrease in viability in the seeded cells precluded comparison with the control group (~ 150 h). The experiments were carried out in biological duplicates. *RBC* red blood cells, *fPD* familial PD, *WT* wild type, *SCDi* stearoyl-coenzyme A desaturase inhibitor

Given the apparent disease and region specificity of the αS^H^ destabilization, and the discussed “prion-like” aggregation transmission of pathological αS in a highly region-specific manner, we analyzed whether the αS^H^ form of αS, being resistant to spontaneous aggregation, can also protect against the self-templating “prion-like” aggregation mediated by αS^F^. Therefore, we performed a thioflavin T-based aggregation assay on purified αS and separated αS^H^ multimers and αS^U^ monomers. As the cross-linker interfered with ThT assays (Online Resource Fig. 15a), we prepared native, non-cross-linked αS^H^ from human blood and αS^U^ from *E. coli*, given the difficulty of native αS purification from brain [38], and assessed its susceptibility toward “prion-like” aggregation. Our ThT-aggregation assay showed that αS^H^—in contrast to monomeric αS^U^—did not form ThT-bound fibrils (Fig. 3a). The addition of recombinant “prion-like” αS^F^ did stimulate the aggregation in the αS^U^ monomer, but not in the αS^H^ multimer, hence indicating that the cytosolic helical structure found in human brain is not susceptible to putative “prion-like” aggregation transmission of amyloid protein species (Fig. 3a). Furthermore, addition of αS^F^ did not lead to destabilization of purified αS^H^. In addition, we analyzed M17D cells with high αS^H^/αS^U^ ratios and found that the addition of αS^F^ to wt cells did not result in a significant decrease of αS^H^/αS^U^ ratios (Online Resource Fig. 15b), indicating that while stabilization of αS^H^ protects from detrimental effects, αS^F^ (amyloid itself) is not causal for destabilization of αS^H^. Taken together, these data suggest that a decrease in αS^H^/αS^U^ ratios potentially leads to a raised susceptibility in neurons for “prion-like” transmission of αS amyloid. To test this, we stimulated “prion-like” aggregation in human neuronal cells (M17D) expressing αS constructs with varying αS^H^/αS^U^ ratios (wt or previously described αS^H^ destabilizing fPD mutations) by addition of preformed αS^F^ to the cell media. We then assessed the amount of (Triton-X) insoluble αS found in the cell pellets by αS ELISA after 2 days of seeding. While no differences in forming insoluble αS were found in cells expressing fPD mutations without treatment (Fig. 3b), the addition of αS^F^ to the growth medium led to a significant increase of aggregated αS in the neuronal cell line across all fPD mutations, indicating an increased “prion-like” aggregation susceptibility compared to wild type (wt, Fig. 3c). This directly indicates that all fPD mutations lead to greater susceptibility toward “prion-like” aggregation in neuronal cells, while their effect on spontaneous aggregation is comparatively small. In accordance with previous published data [14], we detected a decrease of αS^H^/αS^U^ ratio in the lysate of M17D cells expressing different familial PD *SNCA* mutations (A30P, G51D, A53T, E46K and H50Q, respectively) compared to wt (Fig. 3d). The decrease of αS^H^/αS^U^ ratios caused by these mutations correlated significantly with the susceptibility toward “prion-like” aggregation as measured by insoluble αS in αS^F^-seeded M17D cells (*p* = 0.002, Fig. 3d). In contrast, M17D cells with higher αS^H^/αS^U^ ratios exhibited decreased seeding and aggregation capabilities, indicating that increased αS^H^ stabilization in neuronal cells is protective against “prion-like” propagation (Fig. 3d). To establish a causal link between αS^H^ stability and “prion-like” aggregation resistance, we next performed rescue experiments with a stearoyl-coenzyme A desaturase inhibitors (SCDi), a known pharmacological stabilizer of αS multimers [19, 30, 45] leading to an increase in the relative amount of protective αS^H^ in our M17D cells (Fig. 3e). We then seeded M17D cells expressing YFP-tagged wt αS and followed the αS inclusion formation under αS^F^ addition via automated confocal microscopy (IncuCuyte). Here, the multimer-stabilizer SCDi prevents the formation of pathological αS inclusions even in the presence of αS^F^ (Fig. 3f), indicating a rescue of “prion-like” susceptibility through the stabilization of physiologically αS^H^. This protective stabilization even extended to the spontaneous aggregation seen in wt αS expressing neuronal cells without αS^F^ addition (Fig. 3f).

### The disequilibrium of αS^H^ and αS^U^ is brain region specific and associated with dementia in PD and DLB patients

To validate the association between the region-specific distribution and the proposed “prion-like” aggregation transmission theory of αS, we carried out an analysis of nine brain regions from 10 controls, 6 DLB and 13 sporadic PD patients (Table 2). All brain regions for each individual were analyzed in biological and technical duplicates (*n* = 1044 single samples). Compared to the analysis of the four brain regions, which used PD patients mostly at their extremes (Braak 6), we were able to include selected PD patients with Braak stages 4 and 5 and additional clinical information such as the cognitive status or Thal phases (Table 2).

**Table 2:** Information on samples, cross-linking aggregation transmission analysis (9 brain regions)

Case #	PMI [hours]	Age at death [years]	Gender	Disease duration [years]	Dementia	Thal	Braak NFT	CERAD	Braak LB	McKeith
Control 1	79	84	Male		No	0	0	Absent	0	None
Control 2	105	79	Male		No	0	2	Absent	0	None
Control 3	120	86	Female		No	0	2	Absent	0	None
Control 4	60	96	Female		No	2	2	Absent	0	None
Control 5	47	89			No	3	2	Mild	0	None
Control 6	61	101	Male		No	0	1	Absent	0	None
Control 7	79	76	Male		No	1	2	Absent	0	None
Control 8	3	71	Female		No	2	3	Absent	0	None
Control 9	2	84	Female		No	3	2	Mild	0	None
Control 10	2	87	Male		No	2	2	Mild	0	None
DLB 1	45	63	Female	10	Yes		6	Frequent	6	Diffuse neocortical
DLB 2	70	68	Male	13	Yes	5	6	Frequent	6	Diffuse neocortical
DLB 3	63	59	Female	10	Yes	5	3	Moderate	6	Diffuse neocortical
DLB 4	24	60	Male	8	Yes	1	1	Moderate	6	Diffuse neocortical
DLB 5	56	75	Male	16	Yes	1	2	Sparse	6	Diffuse neocortical
DLB 6	55	79	Male	10	Yes	4	2	Sparse	6	Diffuse neocortical
PD 1	114	82	Male	34	No	1	1	Absent	4	Brainstem
PD 2	98	77	Male	8	Yes	1	2	Absent	4	Limbic
PD 3	16	74	Male	13	Yes	1	0	Absent	4	Brainstem
PD 4	99	80	Male	6	No	0	2	Absent	5	Limbic
PD 5	89	86	Female	27	No	0	2	Absent	5	Limbic
PD 6	85	78	Female	11	No	1	1	Absent	5	Limbic
PD 7	35	92	Male	13	No	0	2	Absent	6	Diffuse neocortical
PD 8	24	63	Male	21		0	1	Absent	6	Diffuse neocortical
PD 9	87	76	Female	15	No	0	1	Absent	6	Diffuse neocortical
PD 10	105	85	Female	25	Yes	0	2	Absent	6	Diffuse neocortical
PD 11	100	72	Male	28	Yes	1	2	Absent	6	Diffuse neocortical
PD 12	41	70	Female	39	Yes	0	2	Absent	6	Diffuse neocortical
PD 13	25	70	Female	27	No	1	1	Absent	6	Diffuse neocortical

*PD* Parkinson’s disease, *DLB* dementia with Lewy bodies, *CERAD* Consortium to Establish a Registry for Alzheimer’s Disease, *PMI* post-mortem interval, *LB* Lewy bodies, *NFT* neurofibrillary tangles

**Fig. 4:**
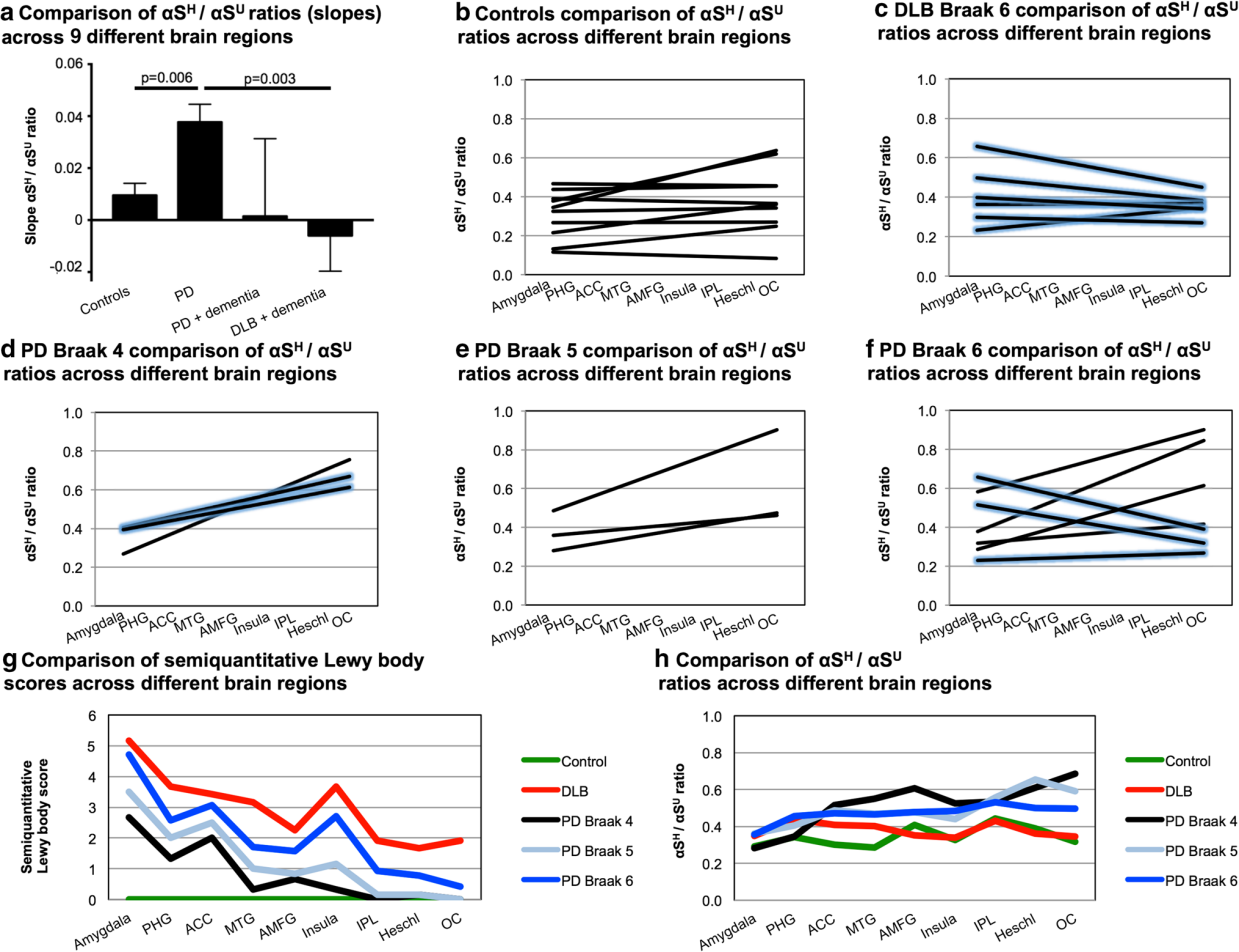
αS^H^/αS^U^ equilibrium is disturbed in PD and DLB patients. For each individual, 9 different brain regions were analyzed, reflecting the temporal development of LB pathology across the limbic and neocortical regions. Amygdala, cortex of the parahippocampal (PHG) and anterior cingulate cortex (ACC) are affected earlier in the disease course, followed by cortex of the insula, middle temporal (MTG), anterior middle frontal gyri (AMFG), and lastly with involvement of the cortex of inferior parietal lobule (IPL). Heschl’s gyrus (Heschl) and cortex of the occipital lobe (OC) are typically spared from LB pathology in PD or involved late in the disease course. Each brain region has been analyzed in biological and technical duplicates and one non-cross-linked control sample. The linear trendlines (slopes) across all nine brain regions is depicted for each individual. **a** Comparison of αS^H^/αS^U^ changes across the nine brain regions comparing controls, PD (Braak 4,5 and 6), PD patients with dementia (Braak 4 and 6) and DLB patients (Braak 6) which were all demented. PD patients exhibit significantly increased slopes compared to controls (*p* = 0.006) and significantly higher slopes than DLB patients (*p* = 0.003). Mean with standard error of the mean (SEM). **b** Individual slopes of all controls across the nine brain regions (*n* = 10). **c** Individual slopes of all DLB patients across the nine brain regions (*n* = 6). DLB patients with dementia are displayed in blue enclosed lines. **d** Individual slopes of PD Braak 4 patients across the nine brain regions (*n* = 3). PD patients with dementia are displayed in blue enclosed lines. **e** Individual slopes of all PD Braak 5 patients across the nine brain regions (*n* = 3). **f** Individual slopes of PD Braak 6 patients across the nine brain regions (*n* = 7). PD patients with dementia are displayed in blue enclosed lines. **g** A semiquantitative LB score demonstrates increased amounts of LBs in later affected brain areas of PD and DLB Braak 6 patients. The mean of the score is displayed (controls *n* = 10, DLB *n* = 6, PD *n* = 13). **h** Comparison of mean αS^H^/αS^U^ changes across the nine brain regions comparing controls, PD and DLB patients (controls *n* = 10, DLB *n* = 6, PD *n* = 13). DLB and PD Braak 6 patients exhibit lower slopes (decreased αS^H^/αS^U^ ratios) in the later affected brain regions

The brain regions selected for the analysis reflect the typical temporal development of LB pathology across the limbic and neocortical regions, with early affected areas [amygdala, cortex of parahippocampal gyrus (PHG) and anterior cingulate cortex (ACC)] in Braak stage 4, later affected areas [cortex of insula, middle temporal (MTG) and anterior middle frontal gyri (AMFG)] corresponding to Braak stage 5 and first-order sensory association area [inferior parietal lobule (IPL)] in Braak stage 6. Also, the cortex of the transverse temporal gyrus (Heschl’s gyrus) and occipital cortex (OC, not collected from primary visual cortex), which are regions typically spared from LB pathology or affected late in disease course, were included in the analysis (Fig. 4). We investigated the changes across all nine brain regions and used the slope or trendline calculated for the regions for further comparisons. Interestingly, in this extended brain region analysis, PD patients had significantly higher slopes across the nine brain regions compared to controls (Fig. 4a, *p* = 0.006). The slopes dropped in PD patients with dementia and further declined in DLB patients which were all reported to be demented compared to PD patients without dementia (Fig. 4, *p* = 0.003). It became obvious that in controls, αS^H^/αS^U^ ratios in the later affected brain areas, according to the classical Braak staging, were not higher compared to PD samples per se (Fig. 4b, d–f). The important changes worked out in the analysis of the nine brain regions is as follows: five of six DLB patients exhibited decreased slopes, meaning lower αS^H^/αS^U^ ratios, in the later affected brain regions according to the classical Braak staging (Fig. 4c). PD patients Braak 4, Braak 5 and PD patients Braak 6 without dementia had increasing slopes, thus higher αS^H^/αS^U^ ratios, especially in the IPL, Heschl’s gyrus and OC (Fig. 4d–f). Interestingly, some PD Braak 6 patients, those with dementia, also showed decreased slopes across the nine brain regions similar to DLB patients resulting in low αS^H^/αS^U^ ratios in the IPL, Heschl’s gyrus and OC (Fig. 4f). We performed a linear regression analysis with all available clinical variables (Table 2) and could support those findings by identifying dementia as the only significant predictor of the outcome variable, the αS^H^/αS^U^ ratios (*p* = 0.03, *R*^2^ = 0.3). Overall, in PD patients Braak 4 and 5 with and without dementia, the slopes across the nine brain regions remained positive with higher αS^H^/αS^U^ ratios in the later affected brain regions (Fig. 4d–e). In PD and DLB patients Braak 6, the slopes clearly separated those patients with (decreased slopes) and without dementia (increased slopes, Fig. 4c, f). Thus, the disequilibrium of αS^H^/αS^U^ ratios in the cortical regions across all nine brain regions seems to be critical for PD and DLB patients. Thus, we assume that the individual equilibrium in PD and DLB patients and especially the cortical αS^H^/αS^U^ ratios are disturbed during the disease. These findings were further supported by applying a semiquantitative LB score across the nine brain regions showing that with increasing Braak stages in PD patients and Braak 6 DLB patients, the LB score increases in the later affected brain regions, especially insula, IPL, Heschl’s gyrus and OC (Fig. 4g). The increased means of the semiquantitative LB score comparing controls, PD and DLB patients was significantly different from the decreasing mean αS^H^/αS^U^ slopes compared across all groups (*p* = 0.02, Fig. 4h). Thus, when the disease progresses and LB scores raise, αS^H^/αS^U^ ratios decline, especially in demented individuals. These findings further support the region-specific distribution and the proposed “prion-like” aggregation transmission theory of αS.

**Fig. 5:**
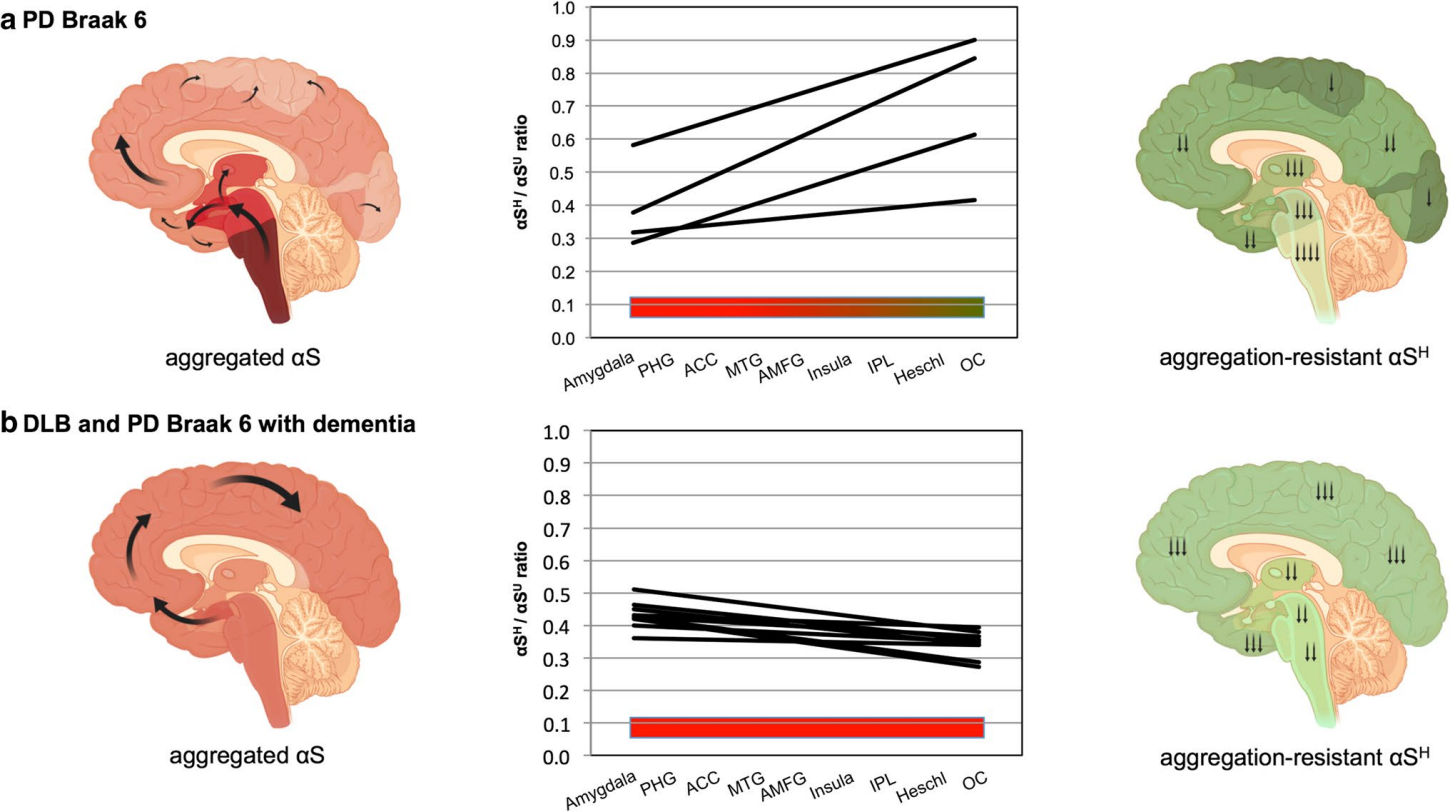
Differences of the αS^H^/αS^U^ equilibriums comparing demented and cognitively intact PD and DLB patients Braak 6. The schematic on the left side depicts the propagation of aggregated αS according to the classical Braak LB stages. For each individual, nine different brain regions were analyzed, reflecting the temporal development of LB pathology across the limbic and neocortical regions. Individual slopes of all patients (PD Braak 6 *n* = 4, PD and DLB with dementia *n* = 9) are shown in the middle schematic. The schematic on the right side shows the proposed decreased amount of αS^H^ in the different brain regions

In summary, when the equilibrium of αS^H^/αS^U^ shifts toward the aggregation-prone αS^U^ in PD and DLB patients, our data demonstrate that the likelihood of fibril formation, and subsequently LB inclusions, increases. Our current findings provide a novel mechanism, in which the equilibrium of physiological aggregation-resistant αS multimers and physiological, aggregation-prone αS monomers is disturbed in sporadic PD and DLB patients, governing the regions affected in the brain and clinical outcome (Fig. 5). Local and individual changes might modify the differential caudo-rostral progression in these synucleinopathies, if the pathology originates in the brain stem as currently described (Fig. 5). The data imply that brain stem pathology would either arrive in neuronal tissue with stepwise increase via midbrain toward the cortices, leading to a slow progression and late involvement of the cortex (PD pattern), or a fast neuropathological progression from brain stem areas to cortical brain regions in DLB patients.

## Discussion

Our study demonstrated that (i) αS^H^ multimers are present in human post-mortem brain tissue and exhibit a physiological, helical secondary structure, making them resistant to spontaneous as well as “prion-like” amyloid aggregation; (ii) the disequilibrium of αS^H^ and αS^U^ is brain region specific and associated with the spreading theory of αS and with clinical features such as dementia. In PD, αS^H^/αS^U^ ratio reflects the regional aggregation transmission of LB pathology as proposed by Braak [8]. In contrast, demented DLB and PD Braak 6 patients exhibit especially low αS^H^/αS^U^ ratios in the neocortical regions. These results are in line with current and previous analysis in familial PD of in vitro and in vivo models on αS^H^, especially the helical αS tetramer [4, 27, 59, 62]. The disequilibrium of all αS^H^ species in fPD models (putative 60 kDa, 80 kDa and 100 kDa after SDS-PAGE) has previously been demonstrated [4, 14, 33, 46]. Our study extends the current knowledge on αS^H^, demonstrating the importance of the αS^H^/αS^U^ equilibrium in healthy human brain tissue and the region-specific disturbance in sporadic PD and DLB patients. Crucially, it provides a potential explanation for region-specific LB pathology in both PD and DLB. Along with these findings, our results indicate that the absolute amount of αS might not be detrimental per se; rather, the equilibrium of physiological aggregation-resistant and aggregation-prone forms of the αS protein, which might be influenced by intrinsic cellular, metabolic and genetic properties of different neuronal subpopulations [1, 57], is important. As already mentioned, we have to recognize that some controls also display lower than average αS^H^/αS^U^ across different brain regions; thus, individual and especially cortical perturbations of the complex αS^H^/αS^U^ equilibrium as well as between free and membrane-bound states may be responsible for the neuropathological and clinical features as well as the progressive neurotoxicity in PD and DLB patients as already discussed elsewhere [20]. These are important factors to consider in the development of effective therapeutic targeting and effective αS biomarker assays. Furthermore, the equilibrium of αS^H^/αS^U^ could be therapeutically shifted by small molecules supporting aggregation resistance in disease-affected cells. Our earlier work [51] showed the role of lipids in stabilizing protective αS^H^, indicating that small molecule design mimicking this lipid-mediated stabilization, or targeting lipid metabolism directly, might be a viable therapeutic approach [19, 30].

Our in vitro data here and a recently published in vivo model [46] indicate a causal role for αS^H^ destabilization in neurodegeneration, but it remains unclear whether further shifts in the equilibrium of αS^H^ and αS^U^ are a part of ongoing progressive pathology in synucleinopathies, maybe further accelerated by the presence of tau or Abeta pathology in the case of DLB. αS^H^ destabilization could possibly result in different pathological αS^F^ conformations in the human patient. In addition, these αS conformations could differentially impact neuronal vulnerability by αS^H^ destabilization in a region-specific manner [1, 17, 32], thereby concluding whether local neuronal vulnerability or “prion-like” aggregation transmission explains disease progression patterns [58]. The answer, implied here by our data, is that both mechanisms coexist, since “prion-like” aggregation and transmission get directed by local vulnerabilities. However, further studies have to answer the question how the equilibrium of αS^H^ and αS^U^ is disturbed during aging and progression of synucleinopathies. Our study mainly focused on limbic and neocortical regions in patients with DLB and PD with most advanced Braak stage 6 neocortical LB pathology. Non-classical PD patients, in which the LB pathology does not follow the classical Braak LB staging, might not show the same disequilibriums of αS^H^/αS^U^. We also did not present data on early affected brain regions, such as nucleus coeruleus, nucleus vagus or substantia nigra, given the low availability of these tissues. Still, we have demonstrated in our neuronal cell model that a general destabilization of αS^H^ is detrimental to aggregation resistance and that stabilizing agents such has SCDi could have a beneficial effect in sporadic PD regardless of the region specificity being applicable to every patient.

Overall, our current findings provide a novel mechanism, in which the equilibrium of physiological aggregation-resistant αS^H^ and physiological aggregation-prone αS^U^ is disturbed in sporadic PD and DLB patients. This brain region-specific pathology suggests pathways that explain progressive aggregation transmission of LB pathology in the human brain along different regions in DLB vs. PD. We propose that a stabilization of physiological aggregation-resistant αS^H^ in PD and DLB patients may be beneficial in slowing down the process of neurodegeneration, analogous to efforts currently underway toward stabilizing transthyretin in familial amyloid polyneuropathy [11, 41]. Beyond therapeutic applications, the ratio of αS^H^/αS^U^ can be used as a biomarker of disease progression.

## Supplementary Information

Below is the link to the electronic supplementary material.

Supplementary file1 (DOCX 13945 kb)

## Data Availability

All data are available in the main text or the supplementary materials.

## References

[1] Alegre-Abarrategui J, Brimblecombe KR, Roberts RF, Velentza-Almpani E, Tilley BS, Bengoa-Vergniory N (2019). Selective vulnerability in α-synucleinopathies. Acta Neuropathol.

[2] Angelova DM, Brown DR (2018). Model senescent microglia induce disease related changes in α-synuclein expression and activity. Biomolecules.

[3] Bargar C, Wang W, Gunzler SA, LeFevre A, Wang Z, Lerner AJ (2021). Streamlined alpha-synuclein RT-QuIC assay for various biospecimens in Parkinson’s disease and dementia with Lewy bodies. Acta Neuropathol Commun.

[4] Bartels T, Choi JG, Selkoe DJ (2011). alpha-Synuclein occurs physiologically as a helically folded tetramer that resists aggregation. Nature.

[5] Baulac S, LaVoie MJ, Strahle J, Schlossmacher MG, Xia W (2004). Dimerization of Parkinson’s disease-causing DJ-1 and formation of high molecular weight complexes in human brain. Mol Cell Neurosci.

[6] Bhumkar A, Magnan C, Lau D, Jun ESW, Dzamko N, Gambin Y (2021). Single-molecule counting coupled to rapid amplification enables detection of α-synuclein aggregates in cerebrospinal fluid of Parkinson’s disease patients. Angew Chem Int Ed Engl.

[7] Bongianni M, Ladogana A, Capaldi S, Klotz S, Baiardi S, Cagnin A (2019). α-Synuclein RT-QuIC assay in cerebrospinal fluid of patients with dementia with Lewy bodies. Ann Clin Transl Neurol.

[8] Braak H, Del TK, Rüb U, de Vos RA, Jansen Steur EN, Braak E (2003). Staging of brain pathology related to sporadic Parkinson’s disease. Neurobiol Aging.

[9] Burré J, Vivona S, Diao J, Sharma M, Brunger AT, Südhof TC (2013). Properties of native brain α-synuclein. Nature.

[10] Candelise N, Schmitz M, Llorens F, Villar-Piqué A, Cramm M, Thom T (2019). Seeding variability of different alpha synuclein strains in synucleinopathies. Ann Neurol.

[11] Coelho LF, Martins da Silva A, Waddington Cruz M, Plante-Bordeneuve V, Lozeron P, Suhr OB (2012). Tafamidis for transthyretin familial amyloid polyneuropathy: a randomized, controlled trial. Neurology.

[12] Dettmer U, Newman AJ, Luth ES, Bartels T, Selkoe D (2013). In vivo cross-linking reveals principally oligomeric forms of α-synuclein and β-synuclein in neurons and non-neural cells. J Biol Chem.

[13] Dettmer U, Newman AJ, von Saucken VE, Bartels T, Selkoe D, Von SVE (2015). KTKEGV repeat motifs are key mediators of normal α -synuclein tetramerization : Their mutation causes excess monomers and neurotoxicity. Proc Natl Acad Sci USA.

[14] Dettmer U, Newman AJ, Soldner F, Luth ES, Kim NC, Von SVE (2015). Parkinson-causing a-synuclein missense mutations shift native tetramers to monomers as amechanism for disease initiation. Nat Commun.

[15] Dettmer U, Ramalingam N, von Saucken VE, Kim T-E, Newman AJ, Terry-Kantor E (2017). Loss of native alpha-synuclein multimerization by strategically mutating its amphipathic helix causes abnormal vesicle interactions in neuronal cells. Hum Mol Genet.

[16] Donadio V, Wang Z, Incensi A, Rizzo G, Fileccia E, Vacchiano V (2021). In vivo diagnosis of synucleinopathies: a comparative study of skin biopsy and RT-QuIC. Neurology.

[17] Engelender S, Isacson O (2017). The threshold theory for Parkinson’s disease. Trends Neurosci.

[18] Fairfoul G, McGuire LI, Pal S, Ironside JW, Neumann J, Christie S (2016). Alpha-synuclein RT-QuIC in the CSF of patients with alpha-synucleinopathies. Ann Clin Transl Neurol.

[19] Fanning S, Haque A, Imberdis T, Baru V, Barrasa MI, Nuber S (2018). Lipidomic analysis of α-synuclein neurotoxicity identifies stearoyl CoA desaturase as a target for parkinson treatment. Mol Cell.

[20] Fanning S, Selkoe D, Dettmer U (2020). Parkinson’s disease: proteinopathy or lipidopathy. NPJ Parkinsons Dis.

[21] Fernandez RD, Lucas HR (2018). Mass spectrometry data confirming tetrameric alpha-synuclein N-terminal acetylation. Data Br.

[22] Fernández RD, Lucas HR (2018). Isolation of recombinant tetrameric N-acetylated α-synuclein. Protein Expr Purif.

[23] Fernández RD, Lucas HR (2018). Mass spectrometry data confirming tetrameric α-synuclein N-terminal acetylation. Data Br.

[24] Garrido A, Fairfoul G, Tolosa ES, Martí MJ, Green A (2019). α-synuclein RT-QuIC in cerebrospinal fluid of LRRK2-linked Parkinson’s disease. Ann Clin Transl Neurol.

[25] Groveman BR, Orrù CD, Hughson AG, Raymond LD, Zanusso G, Ghetti B (2018). Rapid and ultra-sensitive quantitation of disease-associated α-synuclein seeds in brain and cerebrospinal fluid by αSyn RT-QuIC. Acta Neuropathol Commun.

[26] Gurry T, Ullman O, Fisher CK, Perovic I, Pochapsky T, Stultz CM (2013). The dynamic structure of α-synuclein multimers. J Am Chem Soc.

[27] Gurry T, Ullman O, Fisher CK, Perovic I, Pochapsky T, Stultz CM (2013). The dynamic structure of alpha-synuclein multimers. J Am Chem Soc.

[28] Han J-Y, Jang H-S, Green AJE, Choi YP (2020). RT-QuIC-based detection of alpha-synuclein seeding activity in brains of dementia with Lewy Body patients and of a transgenic mouse model of synucleinopathy. Prion.

[29] Imberdis T, Fanning S, Newman A, Ramalingam N, Dettmer U, Bartels T (2019). Studying α-synuclein conformation by intact-cell cross-linking. Alpha-Synuclein methods protoc.

[30] Imberdis T, Negri J, Ramalingam N, Terry-Kantor E, Ho GPH, Fanning S (2019). Cell models of lipid-rich α-synuclein aggregation validate known modifiers of α-synuclein biology and identify stearoyl-CoA desaturase. Proc Natl Acad Sci USA.

[31] Iranzo A, Fairfoul G, Ayudhaya ACN, Serradell M, Gelpi E, Vilaseca I (2021). Detection of α-synuclein in CSF by RT-QuIC in patients with isolated rapid-eye-movement sleep behaviour disorder: a longitudinal observational study. Lancet Neurol.

[32] Jaunmuktane Z, Brandner S (2019). On the journey to uncover the causes of selective cellular and regional vulnerability in neurodegeneration. Acta Neuropathol.

[33] Kim S, Yun SP, Lee S, Umanah GE, Bandaru VVR, Yin X (2018). GBA1 deficiency negatively affects physiological alpha-synuclein tetramers and related multimers. Proc Natl Acad Sci USA.

[34] Kordower JH, Brundin P (2009). Lewy body pathology in long-term fetal nigral transplants: is Parkinson’s disease transmitted from one neural system to another?. Neuropsychopharmacology.

[35] Kordower JH, Chu Y, Hauser RA, Freeman TB, Olanow CW (2008). Lewy body-like pathology in long-term embryonic nigral transplants in Parkinson’s disease. Nat Med.

[36] Lashuel HA, Overk CR, Oueslati A, Masliah E (2013). The many faces of α - synuclein: from structure and toxicity to therapeutic target. Nat Rev Neurosci.

[37] De Luca CMG, Elia AE, Portaleone SM, Cazzaniga FA, Rossi M, Bistaffa E (2019). Efficient RT-QuIC seeding activity for α-synuclein in olfactory mucosa samples of patients with Parkinson’s disease and multiple system atrophy. Transl Neurodegener.

[38] Luth ES, Bartels T, Dettmer U, Kim NC, Selkoe DJ (2015). Purification of α-synuclein from human brain reveals an instability of endogenous multimers as the protein approaches purity. Biochemistry.

[39] Manne S, Kondru N, Hepker M, Jin H, Anantharam V, Lewis M (2019). Ultrasensitive detection of aggregated α-synuclein in glial cells, human cerebrospinal fluid, and brain tissue using the RT-QuIC assay: new high-throughput neuroimmune biomarker assay for parkinsonian disorders. J Neuroimmune Pharmacol.

[40] Manne S, Kondru N, Jin H, Serrano GE, Anantharam V, Kanthasamy A (2020). Blinded RT-QuIC analysis of α-synuclein biomarker in skin tissue from Parkinson’s disease patients. Mov Disord.

[41] Maurer MS, Schwartz JH, Gundapaneni B, Elliott PM, Merlini G, Waddington-Cruz M (2018). Tafamidis treatment for patients with transthyretin amyloid cardiomyopathy. N Engl J Med.

[42] McKeith IG, Dickson DW, Lowe J, Emre M, O’Brien JT, Feldman H (2005). Diagnosis and management of dementia with Lewy bodies: third report of the DLB Consortium. Neurology.

[43] Meade RM, Fairlie DP, Mason JM (2019). Alpha-synuclein structure and Parkinson’s disease – lessons and emerging principles. Mol Neurodegener.

[44] Neupane K, Solanki A, Sosova I, Belov M, Woodside MT (2014). Diverse metastable structures formed by small oligomers of α-synuclein probed by force spectroscopy. PLoS One.

[45] Nuber S, Nam AY, Rajsombath MM, Cirka H, Hronowski X, Wang J (2021). A stearoyl-coenzyme A desaturase inhibitor prevents multiple Parkinson disease phenotypes in α-synuclein mice. Ann Neurol.

[46] Nuber S, Rajsombath M, Minakaki G, Winkler J, Müller CP, Ericsson M (2018). Abrogating native α-synuclein tetramers in mice causes a L-DOPA-responsive motor syndrome closely resembling Parkinson’s disease. Neuron.

[47] Park S-J, Lee Y-J, Park J-H, Jin H-T, Choi M-J, Jung C-G (2021). Establishment of method for the determination of aggregated α-synuclein in DLB patient using RT-QuIC assay. Protein Pept Lett.

[48] Pavlou MAS, Colombo N, Fuertes-Alvarez S, Nicklas S, Cano LG, Marin MC (2019). Diagnosis and management of dementia with Lewy bodies: third report of the DLB Consortium [1]. Acta Neuropathol.

[49] Rajsombath MM, Nam AY, Ericsson M, Nuber S (2019). Female sex and brain-selective estrogen benefit α-synuclein tetramerization and the PD-like motor syndrome in 3K transgenic mice. J Neurosci.

[50] Rossi M, Candelise N, Baiardi S, Capellari S, Giannini G, Orrù CD (2020). Ultrasensitive RT-QuIC assay with high sensitivity and specificity for Lewy body-associated synucleinopathies. Acta Neuropathol.

[51] Rovere M, Sanderson JB, Fonseca-Ornelas L, Patel DS, Bartels T (2018). Refolding of helical soluble alpha-synuclein through transient interaction with lipid interfaces. FEBS Lett.

[52] van Rumund A, Green AJE, Fairfoul G, Esselink RAJ, Bloem BR, Verbeek MM (2019). α-Synuclein real-time quaking-induced conversion in the cerebrospinal fluid of uncertain cases of parkinsonism. Ann Neurol.

[53] Saijo E, Groveman BR, Kraus A, Metrick M, Orrù CD, Hughson AG (2019). Ultrasensitive RT-QuIC seed amplification assays for disease-associated tau, α-synuclein, and prion aggregates. Methods Mol Biol.

[54] Sanderson JB, De S, Jiang H, Rovere M, Jin M, Zaccagnini L (2020). Analysis of α-synuclein species enriched from cerebral cortex of humans with sporadic dementia with Lewy bodies. Brain Commun.

[55] Sano K, Atarashi R, Satoh K, Ishibashi D, Nakagaki T, Iwasaki Y (2018). Prion-like seeding of misfolded α-synuclein in the brains of dementia with Lewy body patients in RT-QUIC. Mol Neurobiol.

[56] Sherer TB, Betarbet R, Greenamyre JT (2001). Pathogenesis of Parkinson’s disease. Curr Opin Investig Drugs.

[57] Tong J, Wong H, Guttman M, Ang LC, Forno LS, Shimadzu M (2010). Brain α-synuclein accumulation in multiple system atrophy, Parkinson’s disease and progressive supranuclear palsy: a comparative investigation. Brain.

[58] Walsh DM, Selkoe DJ (2016). A critical appraisal of the pathogenic protein spread hypothesis of neurodegeneration. Nat Rev Neurosci.

[59] Wang W, Perovic I, Chittuluru J, Kaganovich A, Nguyen LTT, Liao J (2011). A soluble alpha-synuclein construct forms a dynamic tetramer. Proc Natl Acad Sci.

[60] Wang Z, Becker K, Donadio V, Siedlak S, Yuan J, Rezaee M (2020). Skin α-synuclein aggregation seeding activity as a novel biomarker for Parkinson disease. JAMA Neurol.

[61] Xu L, Bhattacharya S, Thompson D (2018). Re-designing the α-synuclein tetramer. Chem Commun.

[62] Xu L, Bhattacharya S, Thompson D (2019). On the ubiquity of helical alpha-synuclein tetramers. Phys Chem Chem Phys.

