# Supplementary material for: Brain region-specific susceptibility of Lewy body pathology in synucleinopathies is governed by α-synuclein conformations
Source: Acta Neuropathol. 2022 Feb 9;143(4):453–69. doi: 10.1007/s00401-022-02406-7 (PMC8960659; doi:10.1007/s00401-022-02406-7)
Supplement: Supplementary file 1 — Supplementary file1 (DOCX 13945 kb) [file 401_2022_2406_MOESM1_ESM.docx]

**Brain-region specific susceptibility of Lewy body pathology in synucleinopathies**

**is governed by α-synuclein conformations.**

**.**

**Authors**

Laura de Boni^1,2^, Aurelia Hays Watson^1^, Ludovica Zaccagnini^1^, Amber Wallis^1^, Kristina Zhelcheska^3^, Nora Kim^4^, John Sanderson^5^, Haiyang Jiang^6^, Elodie Martin^1^, Adam Cantlon^6^, Matteo Rovere^7^, Lei Liu^6^, Marc Sylvester^8^, Tammaryn Lashley^9^, Ulf Dettmer^6^, Zane Jaunmuktane^9,10,11^, Tim Bartels^1*^

Correspondence to: [t.bartels@ucl.ac.uk](mailto:t.bartels@ucl.ac.uk)

**This online resource includes the following files:**

Figures 1 to 15

Table 1


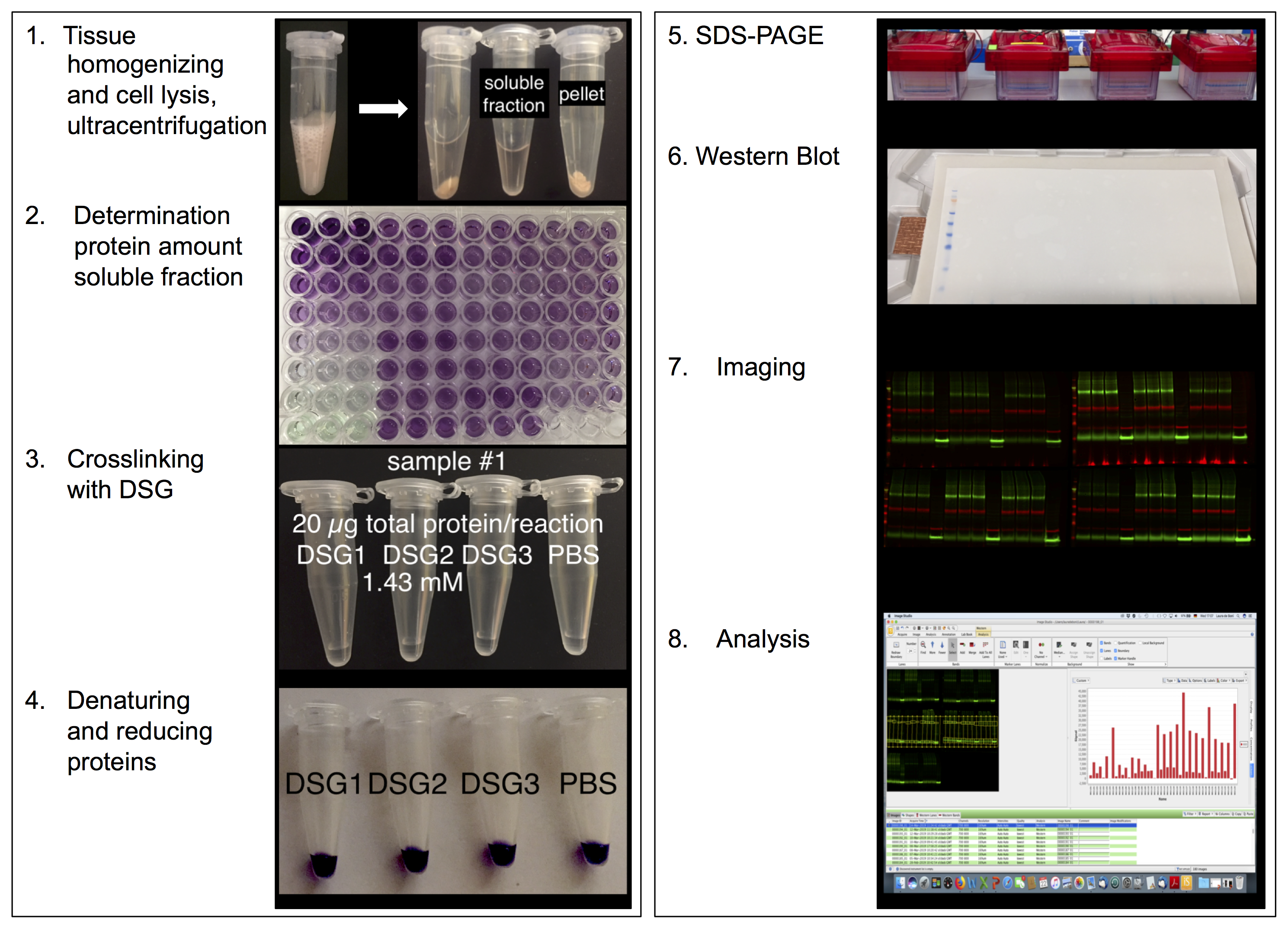
 Fig. 1 Crosslinking protocol for tissue and cell lysates. Protocol and workflow of the crosslinking protocol using tissue and cell lysates. The procedure is described in the Methods section.


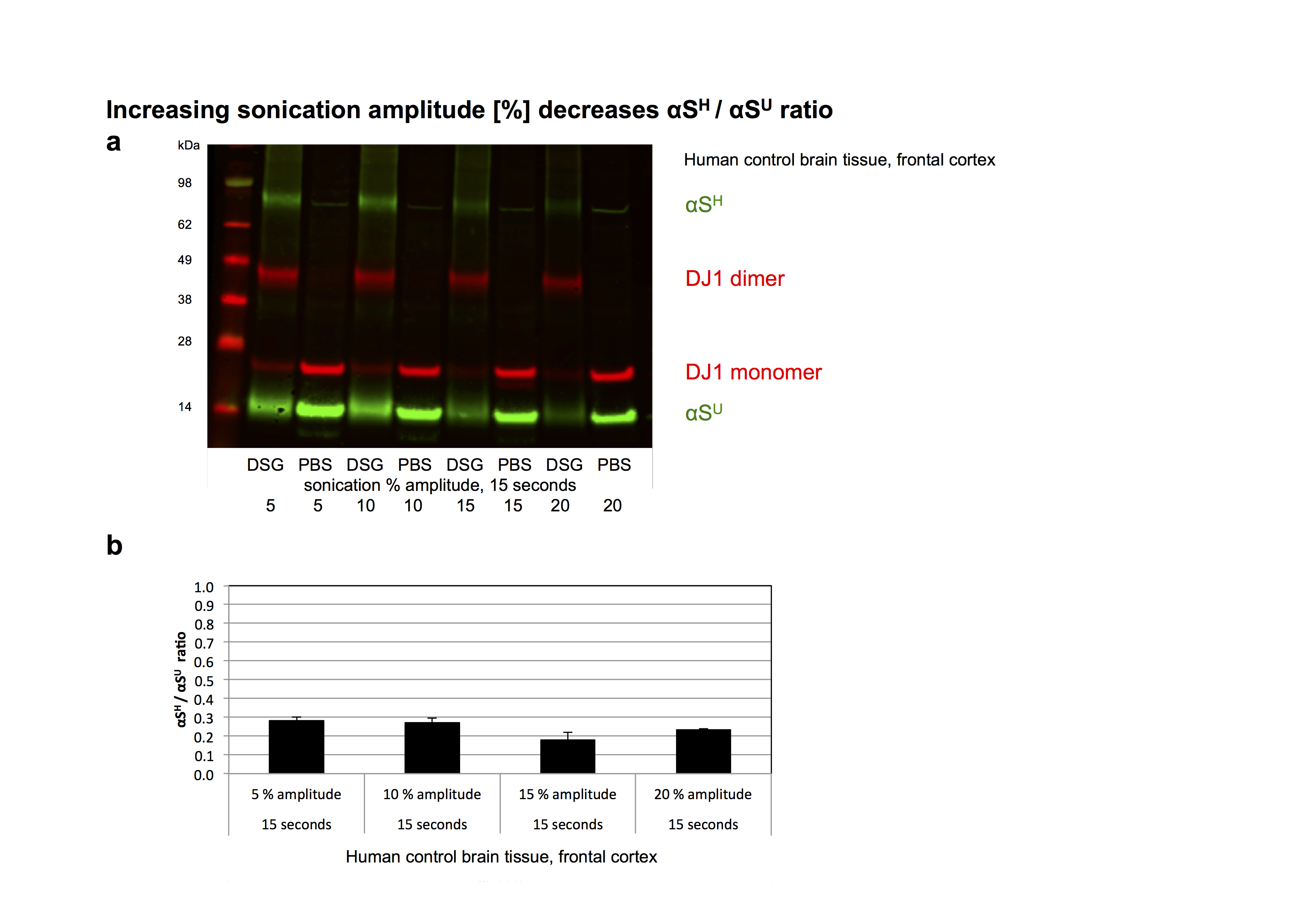


Fig. 2 Validation of the crosslinking protocol. The procedure is described in the Methods section. a Western blot and b quantification of the Western blot of crosslinked (DSG) human control brain tissue (frontal cortex, n=1) subjected to different sonication amplitudes (Fisher Scientific Model 705 Sonic Dismembrator 5 %, 10 %, 15 %, 20 %). Non-crosslinked samples (PBS) are displayed in a. αS^H^ are sensitive to sonication as described previously^1^ and sonication settings were chosen accordingly (Sonic Dismembrator model 300 settings 40, 15 seconds, Fisher Scientific Model 705 Sonic Dismembrator 5 % of amplitude 15 seconds). Green = αS, red = DJ1


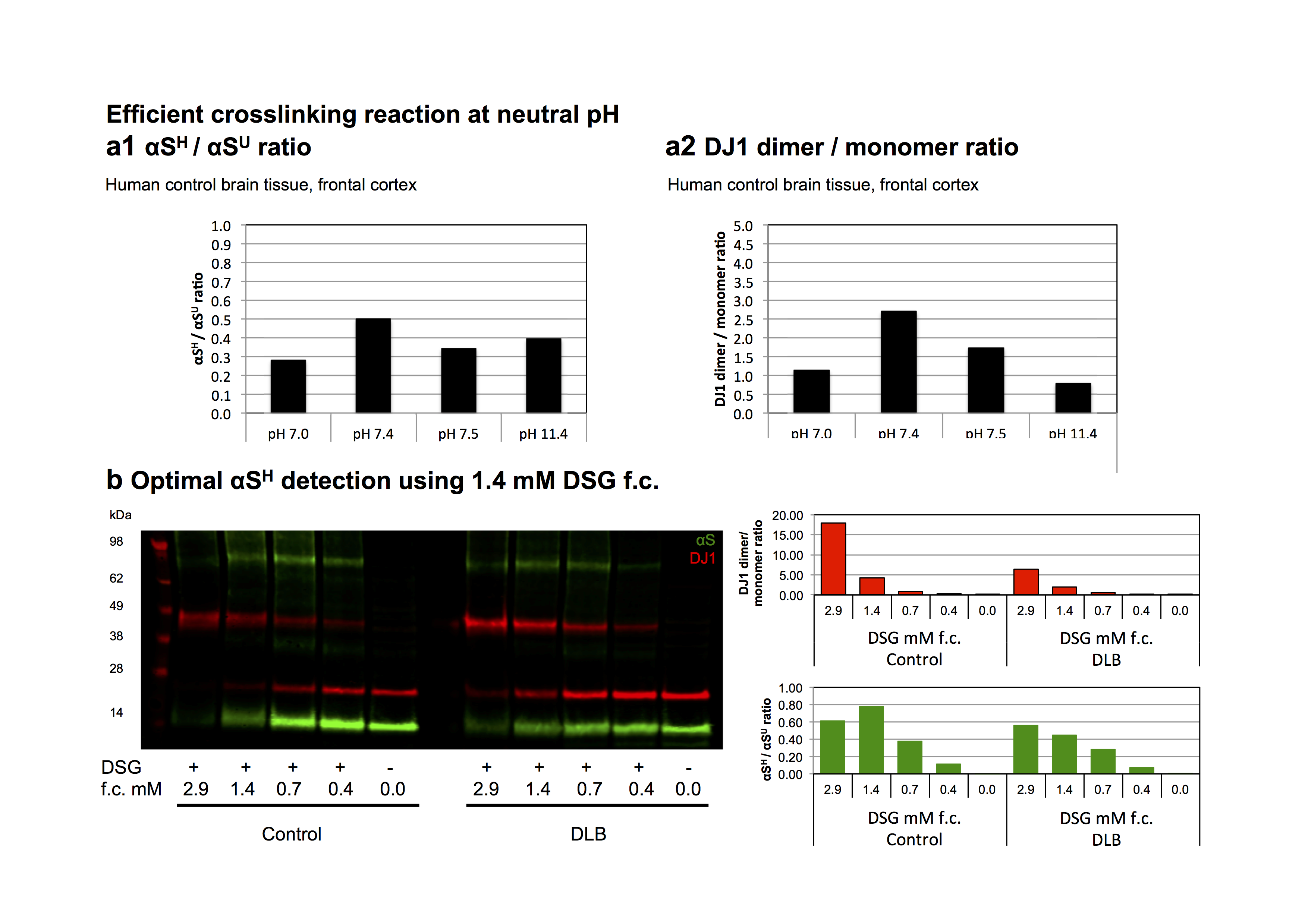


**Fig. 3** Validation of the crosslinking protocol. The procedure is described in the Methods section. **a1** Quantification of αS^H^ / αS^U^ ratios and **a2** DJ1 dimer / monomer ratios in human control brain tissue (frontal cortex, n=1) depending on different pH concentrations. 1x PBS has been used at different pH for solubilization of DSG and for filling up the sample volume to 25 µl total volume. Efficient crosslinking (controlled by DJ1) is carried out at pH 7.4. **b** Western blot with quantifications of crosslinked human control and DLB brain tissue (frontal cortex, n=1 each). The crosslinking reaction is depending on the amount and final concentration of the crosslinker DSG. Thus, for the crosslinking protocol for frozen brain tissue and cell pellets, a final concentration (f.c.) of 1.4 mM DSG was chosen. DSG “+“ = crosslinked sample, DSG “-“ = non-crosslinked (control) sample. Green = αS, red = DJ1


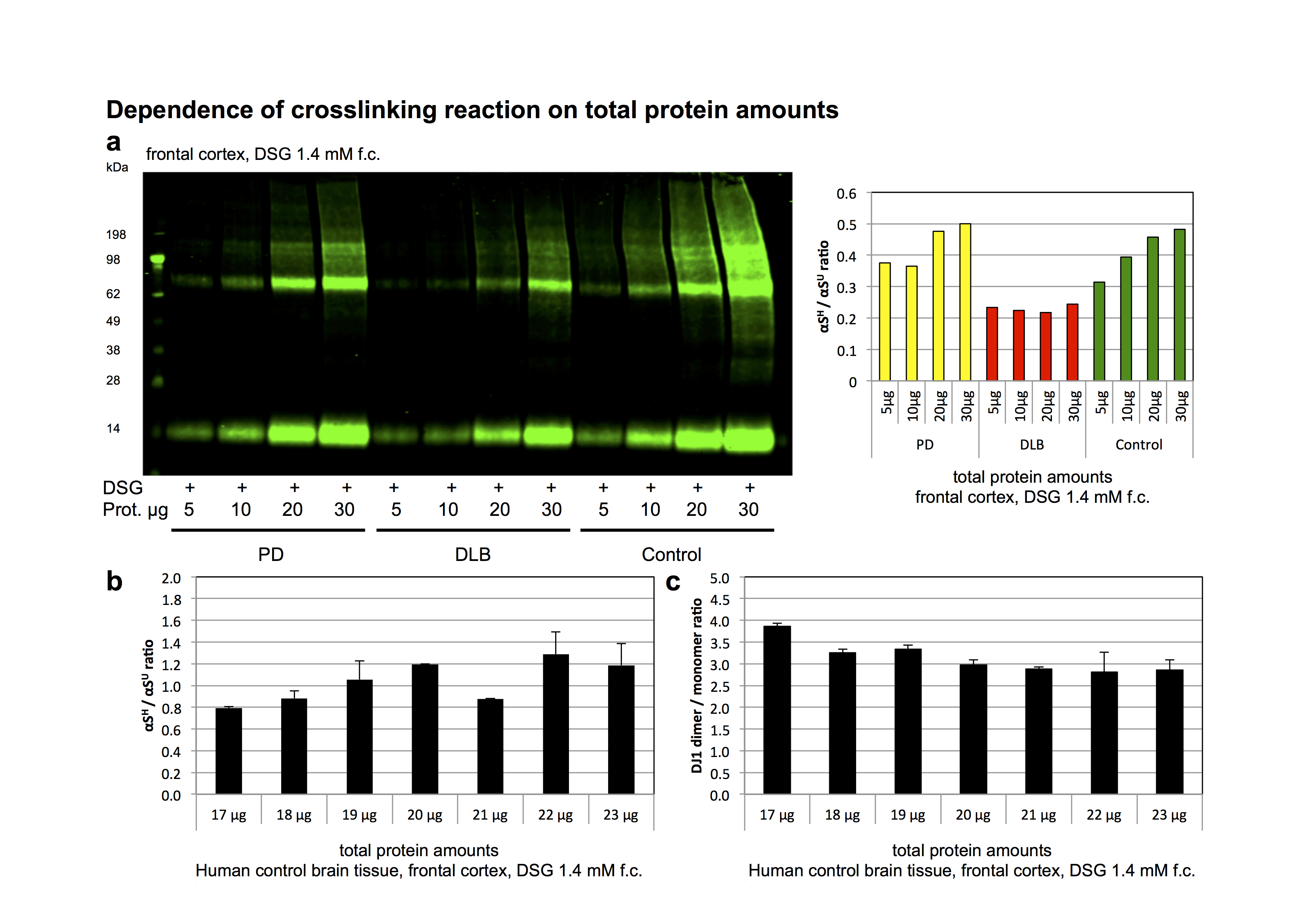


Fig. 4 Validation of the crosslinking protocol. The procedure is described in the Methods section. a Western blot and quantification demonstrating the efficiency of the crosslinking reaction (human brain tissue: control, DLB, PD (n=1 each), frontal cortex) depending on the total amount of protein input (5 µg, 10 µg, 20 µg, 30 µg) into the reaction and the failure to detect higher amounts of αS^H^ in DLB despite a higher total protein input. DSG “+“ = crosslinked sample. b, c Minor changes in total protein input (17 µg, 18 µg, 19 µg, 20 µg, 21 µg, 22 µg, 23 µg) exhibit very similar crosslinking results and efficiency (controlled by DJ1). Human brain tissue, frontal cortex (n=1). F. c. = final concentration.


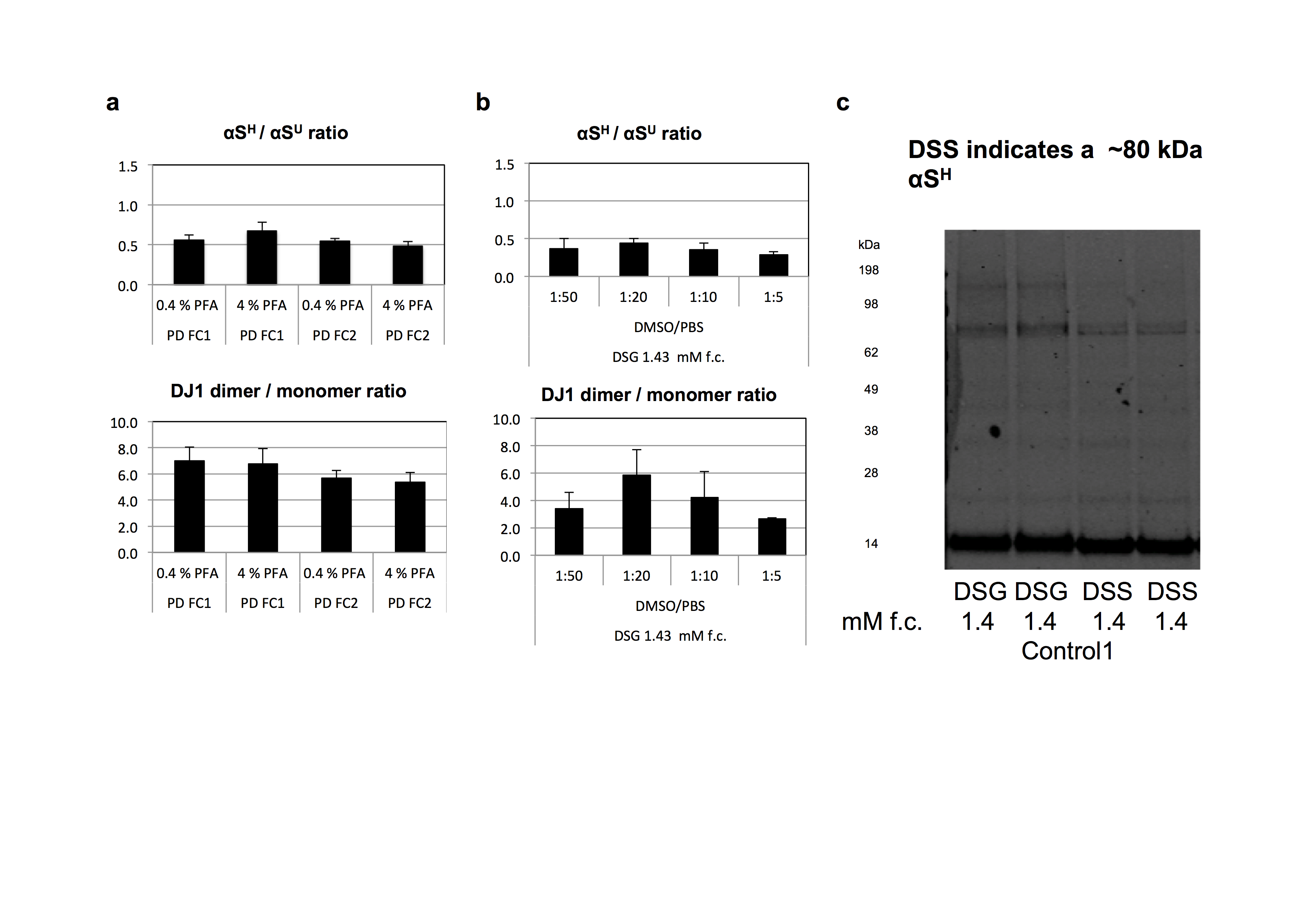


Fig. 5 Validation of the crosslinking protocol. The procedure is described in the Methods section. a Comparison of different PFA concentrations for the fixation of αS. Similar αS^H^ / αS^U^ ratios (A) and DJ1 dimer / monomer ratios upon fixation with PFA 0.4 % or PFA 4 %. Samples have been analyzed in biological duplicates. b DMSO does not lead to an artificial oligomer formation. DSG was solubilized in different amounts of DMSO and added to a protein lysate from frontal cortex control brain tissue. The crosslinking reaction seems to be most efficient at a DSG PBS/DMSO ratio of 1:20. This ratio is used in the crosslinking protocol. PD = Parkinson‘s disease, FC = frontal cortex. c The procedure for the crosslinking is described in the Methods section. Western blot displaying control (n=1) human brain tissue, frontal cortex, crosslinked (technical duplicates) with either DSG or disuccinimidyl suberate (DSS). DSS detects αS^H^ migrating at ~80 kDa in accordance with DSG. All crosslinkers have been used at a final concentration (f.c.) of 1.4 mM in human brain tissue.


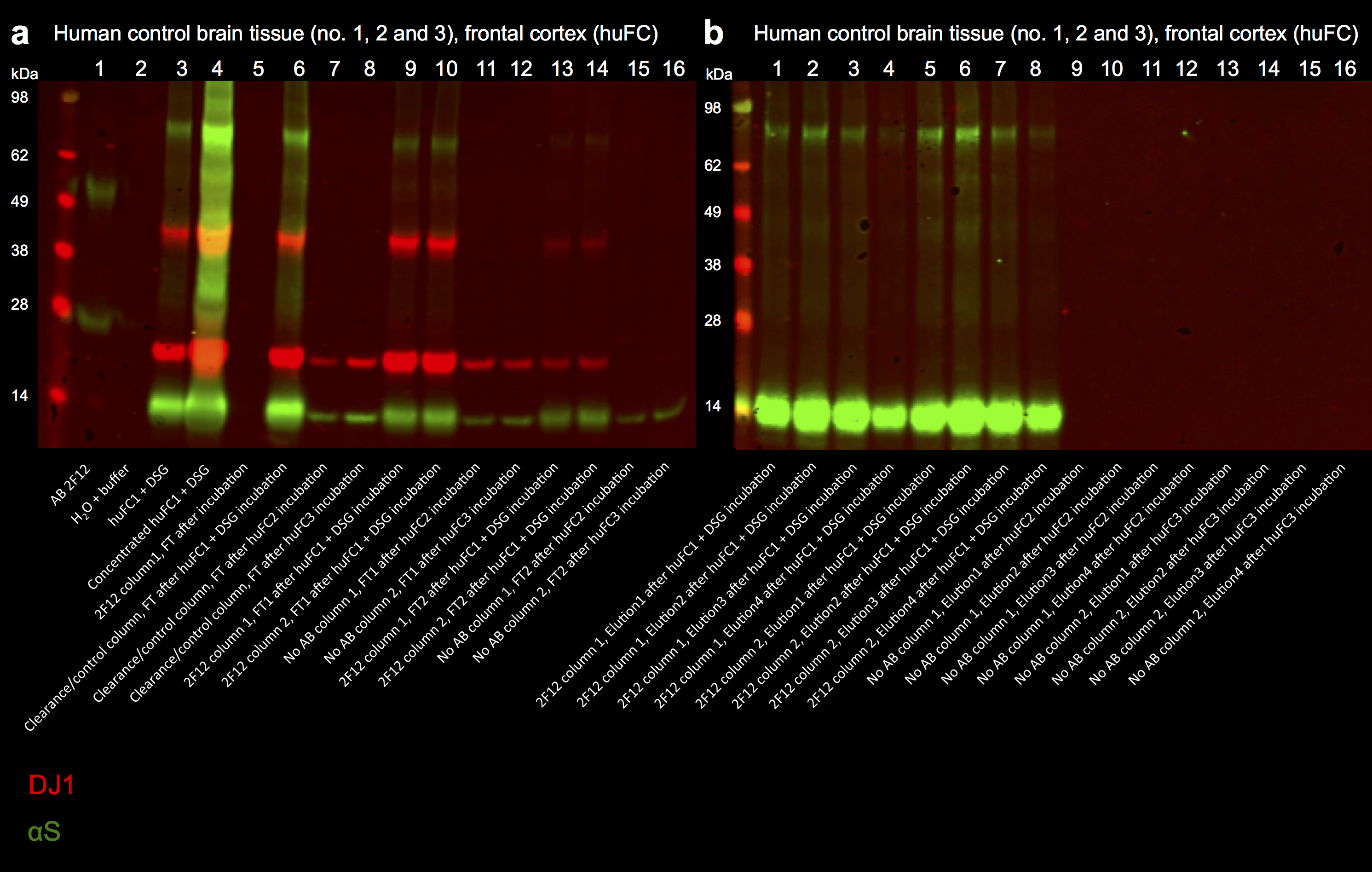


Fig. 6 Immunoprecipitation of αS from human post-mortem native brain tissue. The procedure is described in the Methods section. Samples: 3 different human control brain tissues (no. 1, 2, 3), frontal cortex. The crosslinking procedure is performed as described in the Methods section. a Lane 1: Displaying the applied anti-αS 2F12 antibody (25 kDa light and 50 kDa heavy chain). Lane 2: Used 1x conditioning buffer without artificial protein detection. Lane 3: Crosslinked human brain lysate (no. 1) displaying αS^H^ and αS^U^ (green) and DJ1 dimer and monomer (red). Lane 4: Concentrated crosslinked human brain lysate (no. 1). Lane 5: Flow through (FT) after clearing of the column (AminoLink Plus™ Coupling Resin) incubated with the 2F12 antibody demonstrating complete binding of the 2F12 antibody. Lane 6: FT after incubation of the crosslinked sample no. 1 on the control/clearance column (Pierce Control Agarose Resin, non-amine reactive). Lane 7: FT after incubation of the non-crosslinked sample no. 2 on the control/clearance column. Lane 8: FT after incubation of the non-crosslinked sample no. 3 on the control/clearance column. Lane 9: FT after incubation of the crosslinked sample no. 1 on the 2F12 antibody column. Column is saturated and some protein lost during the washing step. Lane 10: FT after incubation of the crosslinked sample no. 1 on another 2F12 antibody column. Lane 11: FT of column incubated without any antibody and sample no. 2. Lane 12: FT of column incubated without any antibody and sample no. 3. Lane 13: Second FT after incubation of the crosslinked sample no. 1 on the 2F12 antibody column. Column is saturated and some protein lost during the washing step. Lane 14: Second FT after incubation of the crosslinked sample no. 1 on another 2F12 antibody column. Lane 15: Second FT of column incubated without any antibody and sample no. 2. Lane 16: Second FT of column incubated without any antibody and sample no. 3. b Lane 1-4: Elution fractions 1-4 of 2F12 column no. 1 incubated with the crosslinked brain sample no 1 showing the purification of the αS protein (absence of DJ1 protein). Lane 5-8: Elution fractions 1-4 of 2F12 column no. 2 incubated with the crosslinked brain sample no. 1 showing the purification of the αS protein (absence of DJ1 protein). Lane 9-12: Elution fractions 1-4 of one column without antibody incubated with the non-crosslinked brain sample no 2. No protein binding to the column, no elution of protein in the final elution steps. Lane 13-16: Elution fractions 1-4 of a second column without antibody incubated with the non-crosslinked brain sample no 3. No protein binding to the column, no elution of protein in the final elution steps. Green = αS, red = DJ1


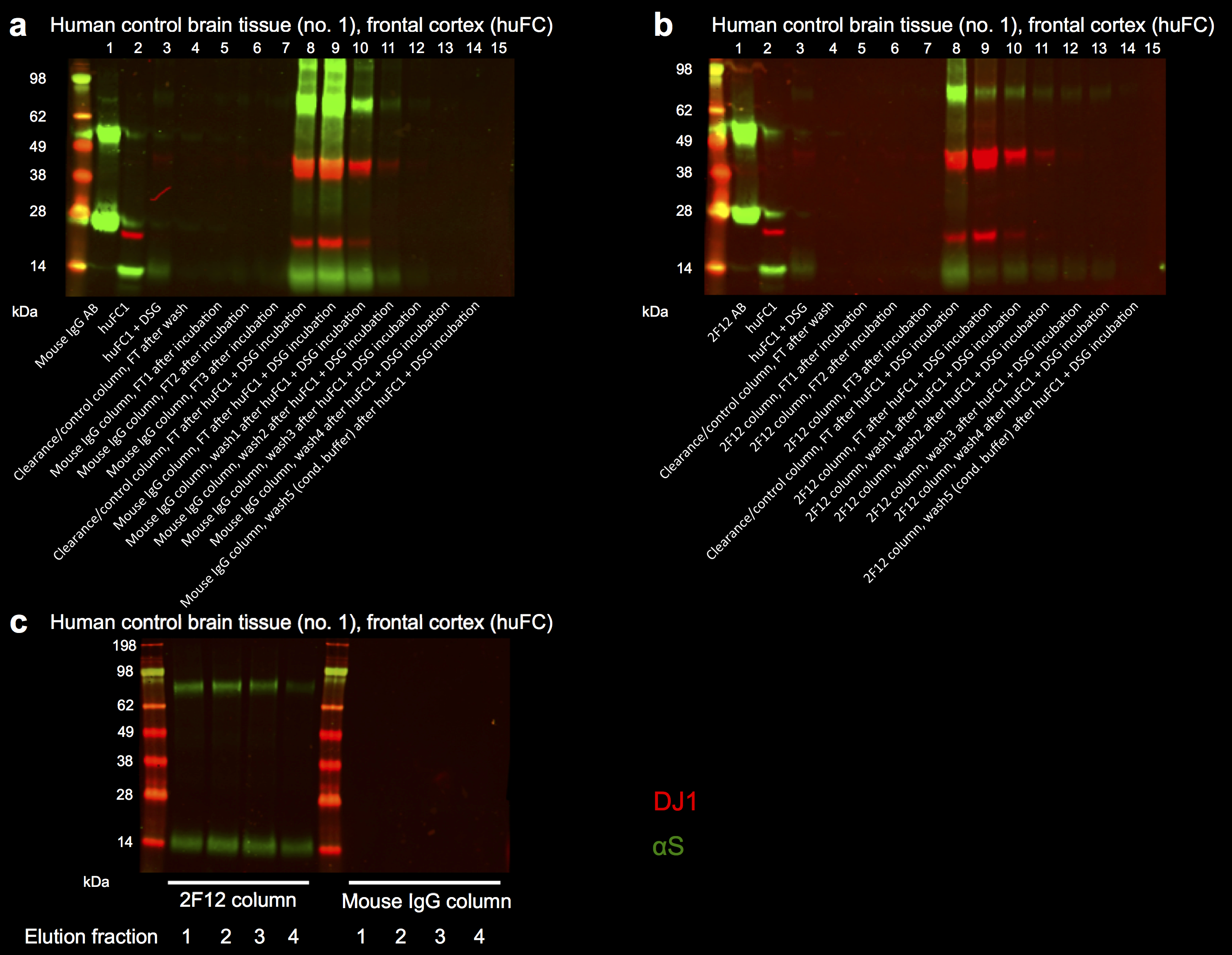


**Fig. 7** Immunoprecipitation of αS from human post-mortem native brain tissue. The procedure is described in the Methods section. Sample: human control brain tissues (no. 1), frontal cortex. The crosslinking is performed according to the procedure described in the Methods section. **a** Lane 1: Displaying the applied mouse IgG (25 kDa light and 50 kDa heavy chain) used as a control antibody. Lane 2: non-crosslinked lysate human brain tissue no. 1. Slight overflow from Lane 1. Lane 3: Crosslinked lysate human brain tissue no. 1. Lane 4: Flow Through (FT) of the washed clearance/control column (Pierce Control Agarose Resin, non-amine reactive); no protein detection. Lane 5-8: FTs after incubation of the antibody column (AminoLink Plus™ Coupling Resin) with the mouse IgG demonstrating complete binding of the mouse IgG. Lane 8: FT after incubation of the clearance/control column with the crosslinked human brain lysate no. 1. Lane 9: FT after incubation of the mouse IgG antibody column with the crosslinked human brain lysate no. 1. Lane 10-15: FT after the washing steps of the mouse IgG antibody column incubated with the crosslinked human brain lysate no. 1. **b** Lane 1: Displaying the applied anti-αS antibody (25 kDa light and 50 kDa heavy chain). Lane 2: Non-crosslinked lysate human brain tissue no. 1. Slight overflow from lane 1. Lane 3: Crosslinked lysate human brain tissue no. 1. Lane 4: Flow Through (FT) of the washed clearance/control column Pierce Control Agarose Resin, non-amine reactive); no protein detection. Lane 5-8: FTs after incubation of the antibody column (AminoLink Plus™ Coupling Resin) with the 2F12 antibody demonstrating complete binding of the 2F12. Lane 8: FT after incubation of the clearance/control column with the crosslinked human brain lysate no. 1. Lane 9: FT after incubation of the 2F12 antibody column with the crosslinked human brain lysate no. 1. Column is saturated, no complete binding of the crosslinked lysate Lane 10-15: FT after the washing steps of the 2F12 antibody column incubated with the crosslinked human brain lysate no. 1. **c** Lane 1-4 2F12 column: Elution fractions 1-4 of 2F12 column incubated with the crosslinked brain sample no 1 showing the purification of the αS protein (absence of DJ1 protein). Lane 1-4 mouse IgG column: Elution fractions 1-4 of mouse IgG column incubated with the crosslinked brain sample no 1 showing no binding and accordingly no elution of αS protein or DJ1. Green = αS, red = DJ1

Table 1 Mass Spectrometry results of purified αS from multiple sources

| **αS sample (gel piece)** | **Accession** | **Description** | **Protein Group IDs** | **Coverage [**  **%]** | **Unique Peptides** | **Protein Unique Peptides** | **AAs** | **MW [kDa]** | **calc. pI** | **Abun--dances** | **Gene Symbol** |
| --- | --- | --- | --- | --- | --- | --- | --- | --- | --- | --- | --- |
| mock 14 kDa | P81605 | Dermicidin | 11 | 20 | 3 | 3 | 110 | 11.3 | 6.54 | 7.3E+06 | DCD |
| mock 14 kDa | Q86YZ3 | Hornerin | 4 | 1 | 2 | 2 | 2850 | 282.2 | 10.04 | 5.7E+06 | HRNR |
| mock 14 kDa | P37840 | α-Synuclein | 12 | 21 | 4 | 2 | 140 | 14.5 | 4.7 | 2.4E+06 | SNCA |
| αS cytosolic unfolded, 14 kDa | P37840 | α-Synuclein | 19 | 40 | 9 | 5 | 140 | 14.5 | 4.7 | 2.8E+07 | SNCA |
| αS cytosolic unfolded, 14 kDa | Q86YZ3 | Hornerin | 5 | 3 | 3 | 3 | 2850 | 282.2 | 10.04 | 2.0E+07 | HRNR |
| αS cytosolic unfolded, 14 kDa | P14923 | Junction plakoglobin | 16 | 4 | 3 | 3 | 745 | 81.7 | 6.14 | 6.9E+06 | JUP |
| αS cytosolic unfolded, 14 kDa | P15924 | Desmoplakin | 13 | 1 | 5 | 5 | 2871 | 331.6 | 6.81 | 3.0E+06 | DSP |
| αS cytosolic unfolded, 14 kDa | P81605 | Dermicidin | 18 | 12 | 3 | 3 | 110 | 11.3 | 6.54 | 1.7E+06 | DCD |
| mock 80 kDa | P37840 | α-Synuclein | 24 | 52 | 12 | 9 | 140 | 14.5 | 4.7 | 1.3E+08 | SNCA |
| mock 80 kDa | Q86YZ3 | Hornerin | 8 | 3 | 2 | 2 | 2850 | 282.2 | 10.04 | 1.7E+07 | HRNR |
| mock 80 kDa | P0DMV8 | Heat shock 70 kDa protein | 21 | 14 | 5 | 1 | 641 | 70 | 5.66 | 7.2E+06 | HSPA1B; HSPA1A |
| mock 80 kDa | P15924 | Desmoplakin | 17 | 1 | 3 | 3 | 2871 | 331.6 | 6.81 | 1.5E+06 | DSP |
| mock 80 kDa | P81605 | Dermicidin | 23 | 21 | 2 | 2 | 110 | 11.3 | 6.54 | 4.9E+05 | DCD |
| αS cytosolic helical, 80 kDa | P0DMV8 | Heat shock 70 kDa protein 1A | 172 | 89 | 43 | 43 | 641 | 70 | 5.66 | 1.6E+11 | HSPA1B; HSPA1A |
| αS cytosolic helical, 80 kDa | P11021 | Endoplasmic reticulum chaperone BiP | 151 | 57 | 40 | 40 | 654 | 72.3 | 5.16 | 1.9E+10 | HSPA5 |
| αS cytosolic helical, 80 kDa | P37840 | α-Synuclein | 200 | 81 | 11 | 9 | 140 | 14.5 | 4.7 | 1.1E+10 | SNCA |
| αS cytosolic helical, 80 kDa | P11142 | Heat shock cognate 71 kDa protein | 128 | 59 | 25 | 25 | 646 | 70.9 | 5.52 | 6.7E+09 | HSPA8 |
| αS cytosolic helical, 80 kDa | P38646 | Stress-70 protein, mitochondrial | 205 | 44 | 25 | 25 | 679 | 73.6 | 6.16 | 2.2E+09 | HSPA9 |
| αS cytosolic helical, 80 kDa | P14136 | Glial fibrillary acidic protein | 32 | 6 | 1 | 1 | 432 | 49.9 | 5.52 | 1.4E+09 | GFAP |
| αS cytosolic helical, 80 kDa | P04406 | Glyceraldehyde-3-phosphate dehydrogenase | 147 | 59 | 14 | 13 | 335 | 36 | 8.46 | 5.2E+08 | GAPDH |
| αS cytosolic helical, 80 kDa | P40926 | Malate dehydrogenase, mitochondrial | 99 | 30 | 7 | 7 | 338 | 35.5 | 8.68 | 3.3E+08 | MDH2 |
| αS cytosolic helical, 80 kDa | P13667 | Protein disulfide-isomerase A4 | 34 | 25 | 13 | 13 | 645 | 72.9 | 5.07 | 3.0E+08 | PDIA4 |
| αS cytosolic helical, 80 kDa | P15924 | Desmoplakin | 113 | 8 | 23 | 23 | 2871 | 331.6 | 6.81 | 1.9E+08 | DSP |
| αS cytosolic helical, 80 kDa | P49915 | GMP synthase | 70 | 15 | 7 | 7 | 693 | 76.7 | 6.87 | 1.6E+08 | GMPS |
| αS cytosolic helical, 80 kDa | Q5D862 | Filaggrin-2 | 106 | 2 | 5 | 5 | 2391 | 247.9 | 8.31 | 1.6E+08 | FLG2 |
| αS cytosolic helical, 80 kDa | P31944 | Caspase-14 | 5 | 30 | 7 | 7 | 242 | 27.7 | 5.58 | 1.5E+08 | CASP14 |
| αS cytosolic helical, 80 kDa | Q12931 | Heat shock protein 75 kDa, mitochondrial | 112 | 17 | 8 | 8 | 704 | 80.1 | 8.21 | 1.5E+08 | TRAP1 |
| αS cytosolic helical, 80 kDa | Q02413 | Desmoglein-1 | 108 | 17 | 12 | 12 | 1049 | 113.7 | 5.03 | 1.4E+08 | DSG1 |
| αS cytosolic helical, 80 kDa | P07237 | Protein disulfide-isomerase | 96 | 31 | 11 | 11 | 508 | 57.1 | 4.87 | 1.0E+08 | P4HB |
| αS cytosolic helical, 80 kDa | P10809 | 60 kDa heat shock protein, mitochondrial | 207 | 21 | 7 | 7 | 573 | 61 | 5.87 | 8.6E+07 | HSPD1 |
| αS cytosolic helical, 80 kDa | Q5T750 | Skin-specific protein 32 | 211 | 4 | 2 | 2 | 250 | 26.2 | 7.97 | 7.5E+07 | C1orf68 |
| αS cytosolic helical, 80 kDa | P17066 | Heat shock 70 kDa protein 6 | 75 | 22 | 3 | 3 | 643 | 71 | 6.14 | 7.1E+07 | HSPA6 |
| αS cytosolic helical, 80 kDa | P08133 | Annexin A6 | 11 | 27 | 15 | 15 | 673 | 75.8 | 5.6 | 7.1E+07 | ANXA6 |
| αS cytosolic helical, 80 kDa | P07355 | Annexin A2 | 145 | 37 | 12 | 4 | 339 | 38.6 | 7.75 | 6.5E+07 | ANXA2 |
| αS cytosolic helical, 80 kDa | Q08554 | Desmocollin-1 | 246 | 7 | 5 | 5 | 894 | 99.9 | 5.43 | 5.2E+07 | DSC1 |
| αS cytosolic helical, 80 kDa | P07195 | L-lactate dehydrogenase B chain | 137 | 22 | 7 | 7 | 334 | 36.6 | 6.05 | 5.1E+07 | LDHB |
| αS cytosolic helical, 80 kDa | P50395 | Rab GDP dissociation inhibitor beta | 226 | 28 | 5 | 5 | 445 | 50.6 | 6.47 | 5.1E+07 | GDI2 |
| αS cytosolic helical, 80 kDa | Q15181 | Inorganic pyrophosphatase | 63 | 21 | 4 | 4 | 289 | 32.6 | 5.86 | 5.0E+07 | PPA1 |
| αS cytosolic helical, 80 kDa | Q15084 | Protein disulfide-isomerase A6 | 210 | 9 | 2 | 2 | 440 | 48.1 | 5.08 | 4.7E+07 | PDIA6 |
| αS cytosolic helical, 80 kDa | P14923 | Junction plakoglobin | 153 | 12 | 8 | 7 | 745 | 81.7 | 6.14 | 4.5E+07 | JUP |
| αS cytosolic helical, 80 kDa | P07900 | Heat shock protein HSP 90-alpha | 43 | 15 | 5 | 4 | 732 | 84.6 | 5.02 | 4.3E+07 | HSP90AA1 |
| αS cytosolic helical, 80 kDa | P17812 | CTP synthase 1 | 248 | 17 | 8 | 8 | 591 | 66.6 | 6.46 | 4.1E+07 | CTPS1 |
| αS cytosolic helical, 80 kDa | Q92945 | Far upstream element-binding protein 2 | 146 | 3 | 2 | 2 | 711 | 73.1 | 7.3 | 4.0E+07 | KHSRP |
| αS cytosolic helical, 80 kDa | P14618 | Pyruvate kinase PKM | 222 | 17 | 6 | 5 | 531 | 57.9 | 7.84 | 3.5E+07 | PKM |
| αS cytosolic helical, 80 kDa | P23526 | Adenosylhomocysteinase | 184 | 9 | 4 | 4 | 432 | 47.7 | 6.34 | 3.4E+07 | AHCY |
| αS cytosolic helical, 80 kDa | P00338 | L-lactate dehydrogenase A chain | 171 | 14 | 4 | 4 | 332 | 36.7 | 8.27 | 3.3E+07 | LDHA |
| αS cytosolic helical, 80 kDa | P29401 | Transketolase | 102 | 12 | 5 | 5 | 623 | 67.8 | 7.66 | 2.9E+07 | TKT |
| αS cytosolic helical, 80 kDa | P19623 | Spermidine synthase | 141 | 8 | 3 | 3 | 302 | 33.8 | 5.49 | 2.6E+07 | SRM |
| αS cytosolic helical, 80 kDa | P13797 | Plastin-3 | 203 | 17 | 9 | 6 | 630 | 70.8 | 5.6 | 2.6E+07 | PLS3 |
| αS cytosolic helical, 80 kDa | P40925 | Malate dehydrogenase, cytoplasmic | 216 | 13 | 3 | 3 | 334 | 36.4 | 7.36 | 2.6E+07 | MDH1 |
| αS cytosolic helical, 80 kDa | Q13867 | Bleomycin hydrolase | 193 | 5 | 2 | 2 | 455 | 52.5 | 6.27 | 2.6E+07 | BLMH |
| αS cytosolic helical, 80 kDa | P00491 | Purine nucleoside phosphorylase | 185 | 34 | 7 | 7 | 289 | 32.1 | 6.95 | 2.5E+07 | PNP |
| αS cytosolic helical, 80 kDa | P05089 | Arginase-1 | 158 | 22 | 5 | 5 | 322 | 34.7 | 7.21 | 2.2E+07 | ARG1 |
| αS cytosolic helical, 80 kDa | P06733 | Alpha-enolase | 206 | 14 | 4 | 3 | 434 | 47.1 | 7.39 | 2.1E+07 | ENO1 |
| αS cytosolic helical, 80 kDa | P14625 | Endoplasmin | 15 | 11 | 6 | 4 | 803 | 92.4 | 4.84 | 2.1E+07 | HSP90B1 |
| αS cytosolic helical, 80 kDa | P54652 | Heat shock-related 70 kDa protein 2 | 92 | 38 | 8 | 8 | 639 | 70 | 5.74 | 2.0E+07 | HSPA2 |
| αS cytosolic helical, 80 kDa | P78371 | T-complex protein 1 subunit beta | 174 | 13 | 4 | 4 | 535 | 57.5 | 6.46 | 1.9E+07 | CCT2 |
| αS cytosolic helical, 80 kDa | Q96QA5 | Gasdermin-A | 230 | 9 | 3 | 3 | 445 | 49.3 | 5.29 | 1.8E+07 | GSDMA |
| αS cytosolic helical, 80 kDa | Q96AE4 | Far upstream element-binding protein 1 | 16 | 7 | 3 | 3 | 644 | 67.5 | 7.61 | 1.8E+07 | FUBP1 |
| αS cytosolic helical, 80 kDa | P36551 | Oxygen-dependent coproporphyrinogen-III oxidase, mitochondrial | 215 | 11 | 4 | 4 | 454 | 50.1 | 8.25 | 1.7E+07 | CPOX |
| αS cytosolic helical, 80 kDa | P48147 | Prolyl endopeptidase OS=Homo sapiens | 67 | 4 | 2 | 2 | 710 | 80.6 | 5.86 | 1.5E+07 | PREP |
| αS cytosolic helical, 80 kDa | P22234 | Multifunctional protein ADE2 OS=Homo sapiens | 115 | 8 | 2 | 2 | 425 | 47 | 7.23 | 1.5E+07 | PAICS |
| αS cytosolic helical, 80 kDa | P38117 | Electron transfer flavoprotein subunit beta | 170 | 7 | 2 | 2 | 255 | 27.8 | 8.1 | 1.4E+07 | ETFB |
| αS cytosolic helical, 80 kDa | P13639 | Elongation factor 2 | 179 | 10 | 6 | 6 | 858 | 95.3 | 6.83 | 1.3E+07 | EEF2 |
| αS cytosolic helical, 80 kDa | P07384 | Calpain-1 catalytic subunit | 238 | 6 | 3 | 3 | 714 | 81.8 | 5.67 | 1.3E+07 | CAPN1 |
| αS cytosolic helical, 80 kDa | P26038 | Moesin | 116 | 14 | 4 | 4 | 577 | 67.8 | 6.4 | 1.3E+07 | MSN |
| αS cytosolic helical, 80 kDa | P08238 | Heat shock protein HSP 90-beta | 101 | 15 | 4 | 2 | 724 | 83.2 | 5.03 | 1.2E+07 | HSP90AB1 |
| αS cytosolic helical, 80 kDa | P62258 | 14-3-3 protein epsilon | 247 | 13 | 3 | 2 | 255 | 29.2 | 4.74 | 1.2E+07 | YWHAE |
| αS cytosolic helical, 80 kDa | O95302 | Peptidyl-prolyl cis-trans isomerase FKBP9 | 103 | 3 | 2 | 2 | 570 | 63 | 5.08 | 1.2E+07 | FKBP9 |
| αS cytosolic helical, 80 kDa | P06576 | ATP synthase subunit beta, mitochondrial | 167 | 11 | 4 | 4 | 529 | 56.5 | 5.4 | 1.1E+07 | ATP5B |
| αS cytosolic helical, 80 kDa | P32119 | Peroxiredoxin-2 OS=Homo sapiens | 244 | 27 | 3 | 3 | 198 | 21.9 | 5.97 | 1.1E+07 | PRDX2 |
| αS cytosolic helical, 80 kDa | P15311 | Ezrin | 127 | 11 | 3 | 3 | 586 | 69.4 | 6.27 | 1.1E+07 | EZR |
| αS cytosolic helical, 80 kDa | Q04837 | Single-stranded DNA-binding protein, mitochondrial | 39 | 16 | 2 | 2 | 148 | 17.2 | 9.6 | 1.1E+07 | SSBP1 |
| αS cytosolic helical, 80 kDa | Q9BWD1 | Acetyl-CoA acetyltransferase, cytosolic | 132 | 14 | 3 | 3 | 397 | 41.3 | 6.92 | 1.0E+07 | ACAT2 |
| αS cytosolic helical, 80 kDa | P00505 | Aspartate aminotransferase, mitochondrial | 220 | 8 | 3 | 3 | 430 | 47.5 | 9.01 | 1.0E+07 | GOT2 |
| αS cytosolic helical, 80 kDa | P41250 | Glycine--tRNA ligase | 240 | 9 | 4 | 4 | 739 | 83.1 | 7.03 | 9.4E+06 | GARS |
| αS cytosolic helical, 80 kDa | P19338 | Nucleolin | 178 | 11 | 6 | 6 | 710 | 76.6 | 4.7 | 8.2E+06 | NCL |
| αS cytosolic helical, 80 kDa | Q00839 | Heterogeneous nuclear ribonucleoprotein U | 136 | 4 | 2 | 2 | 825 | 90.5 | 6 | 7.8E+06 | HNRNPU |
| αS cytosolic helical, 80 kDa | Q99798 | Aconitate hydratase, mitochondrial | 225 | 4 | 2 | 2 | 780 | 85.4 | 7.61 | 7.8E+06 | ACO2 |
| αS cytosolic helical, 80 kDa | P34932 | Heat shock 70 kDa protein 4 | 129 | 6 | 4 | 4 | 840 | 94.3 | 5.19 | 7.8E+06 | HSPA4 |
| αS cytosolic helical, 80 kDa | P26639 | Threonine--tRNA ligase, cytoplasmic | 148 | 3 | 2 | 2 | 723 | 83.4 | 6.67 | 7.8E+06 | TARS |
| αS cytosolic helical, 80 kDa | Q96P63 | Serpin B12 OS=Homo sapiens | 152 | 11 | 4 | 4 | 405 | 46.2 | 5.53 | 7.8E+06 | SERPINB12 |
| αS cytosolic helical, 80 kDa | P30101 | Protein disulfide-isomerase A3 | 159 | 4 | 2 | 2 | 505 | 56.7 | 6.35 | 7.4E+06 | PDIA3 |
| αS cytosolic helical, 80 kDa | P61604 | 10 kDa heat shock protein, mitochondrial | 114 | 29 | 3 | 3 | 102 | 10.9 | 8.92 | 7.3E+06 | HSPE1 |
| αS cytosolic helical, 80 kDa | Q01813 | ATP-dependent 6-phosphofructokinase, platelet type | 104 | 6 | 2 | 1 | 784 | 85.5 | 7.55 | 5.1E+06 | PFKP |
| αS cytosolic helical, 80 kDa | P26641 | Elongation factor 1-gamma | 4 | 5 | 2 | 2 | 437 | 50.1 | 6.67 | 5.0E+06 | EEF1G |
| αS cytosolic helical, 80 kDa | O00154 | Cytosolic acyl coenzyme A thioester hydrolase | 224 | 6 | 2 | 2 | 380 | 41.8 | 8.54 | 4.9E+06 | ACOT7 |
| αS cytosolic helical, 80 kDa | Q96P16 | Regulation of nuclear pre-mRNA domain-containing protein 1A | 27 | 5 | 2 | 2 | 312 | 35.7 | 7.55 | 4.8E+06 | RPRD1A |
| αS cytosolic helical, 80 kDa | P31150 | Rab GDP dissociation inhibitor alpha | 62 | 14 | 1 | 1 | 447 | 50.6 | 5.14 | 4.8E+06 | GDI1 |
| αS cytosolic helical, 80 kDa | Q9UI42 | Carboxypeptidase A4 | 9 | 7 | 2 | 2 | 421 | 47.3 | 6.7 | 4.7E+06 | CPA4 |
| αS cytosolic helical, 80 kDa | P50991 | T-complex protein 1 subunit delta | 38 | 5 | 2 | 2 | 539 | 57.9 | 7.83 | 4.2E+06 | CCT4 |
| αS cytosolic helical, 80 kDa | P22735 | Protein-glutamine gamma-glutamyltransferase K | 30 | 5 | 3 | 3 | 817 | 89.7 | 6.04 | 4.0E+06 | TGM1 |
| αS cytosolic helical, 80 kDa | Q08188 | Protein-glutamine gamma-glutamyltransferase E | 23 | 13 | 8 | 8 | 693 | 76.6 | 5.86 | 3.9E+06 | TGM3 |
| αS cytosolic helical, 80 kDa | P37837 | Transaldolase OS=Homo sapiens | 202 | 7 | 2 | 2 | 337 | 37.5 | 6.81 | 3.8E+06 | TALDO1 |
| αS cytosolic helical, 80 kDa | P08670 | Vimentin | 41 | 5 | 1 | 1 | 466 | 53.6 | 5.12 | 3.8E+06 | VIM |
| αS cytosolic helical, 80 kDa | Q8NBF2 | NHL repeat-containing protein 2 | 255 | 7 | 4 | 4 | 726 | 79.4 | 5.55 | 3.5E+06 | NHLRC2 |
| αS cytosolic helical, 80 kDa | P25786 | Proteasome subunit alpha type-1 | 60 | 10 | 2 | 2 | 263 | 29.5 | 6.61 | 3.3E+06 | PSMA1 |
| αS cytosolic helical, 80 kDa | P09960 | Leukotriene A-4 hydrolase | 236 | 6 | 2 | 2 | 611 | 69.2 | 6.18 | 2.8E+06 | LTA4H |
| αS cytosolic helical, 80 kDa | O43175 | D-3-phosphoglycerate dehydrogenase | 66 | 6 | 3 | 3 | 533 | 56.6 | 6.71 | 2.5E+06 | PHGDH |
| αS cytosolic helical, 80 kDa | P16278 | Beta-galactosidase | 76 | 5 | 3 | 3 | 677 | 76 | 6.57 | 2.2E+06 | GLB1 |
| αS cytosolic helical, 80 kDa | O95757 | Heat shock 70 kDa protein 4L | 74 | 3 | 2 | 2 | 839 | 94.5 | 5.88 | 1.6E+06 | HSPA4L |
| E. coli recombinant αS 14 kDa | P0DMV8 | Heat shock 70 kDa protein 1A | 23 | 49 | 34 | 17 | 641 | 70 | 5.66 | 2.6E+09 | HSPA1B; HSPA1A |
| E. coli recombinant αS 14 kDa | P37840 | Alpha-synuclein | 26 | 69 | 16 | 12 | 140 | 14.5 | 4.7 | 1.3E+09 | SNCA |
| E. coli recombinant αS 14 kDa | P11021 | Endoplasmic reticulum chaperone BiP | 20 | 19 | 9 | 9 | 654 | 72.3 | 5.16 | 2.0E+08 | HSPA5 |
| E. coli recombinant αS 14 kDa | P11142 | Heat shock cognate 71 kDa protein | 18 | 22 | 7 | 5 | 646 | 70.9 | 5.52 | 6.1E+07 | HSPA8 |
| E. coli recombinant αS 14 kDa | P38646 | Stress-70 protein, mitochondrial | 27 | 4 | 3 | 3 | 679 | 73.6 | 6.16 | 2.8E+07 | HSPA9 |
| E. coli recombinant αS 14 kDa | P0ACF8 | DNA-binding protein H-NS | 29 | 15 | 3 | 3 | 137 | 15.5 | 5.47 | 1.6E+07 | hns |
| E. coli recombinant αS 14 kDa | P02452 | Collagen alpha-1(I) chain | 15 | 1 | 2 | 2 | 1464 | 138.9 | 5.8 | 9.1E+06 | COL1A1 |
| E. coli recombinant αS 14 kDa | Q86YZ3 | Hornerin | 8 | 3 | 2 | 2 | 2850 | 282.2 | 10.04 | 4.1E+06 | HRNR |
| E. coli recombinant αS 14 kDa | P08123 | Collagen alpha-2(I) chain | 22 | 1 | 2 | 2 | 1366 | 129.2 | 8.95 | 3.0E+06 | COL1A2 |

Mass spectrometry data of αS from HEK. The detailed procedure is described in the methods section. HEK293 αS C-term Strep II-tagged lysates were crosslinked and αS^H^ and αS^U^ were immunoprecipitated using StrepTrap 5ml columns. αS^H^ and αS^U^ were separated via size exclusion chromatography, run on a SDS-gel and gel pieces were lyophilized prior Mass spectrometry analysis. Lyophilized gel pieces were digested with trypsin. The quantification analysis was based on protein-specific peptides. For the mock sample, regular HEK293 were used. To further exclude false positive hits, recombinant αS^U^ was analyzed as well. The mock αS from regular HEK has a low abundance of αS, depicting αS^U^ remnants from HEK trapped in the StrepTap column. The αS from the 80 kDa gel piece exhibits 100 times more αS than the mock 80 kDa sample and the false discovery rate of proteins in these samples is 0.6 %. The E. coli 14 kDa band samples show several false positive hits including human HSP70. The data show that the most abundant protein in the αS^H^ sample except confirmed false positives is αS, strongly indicating a homo-oligomer.


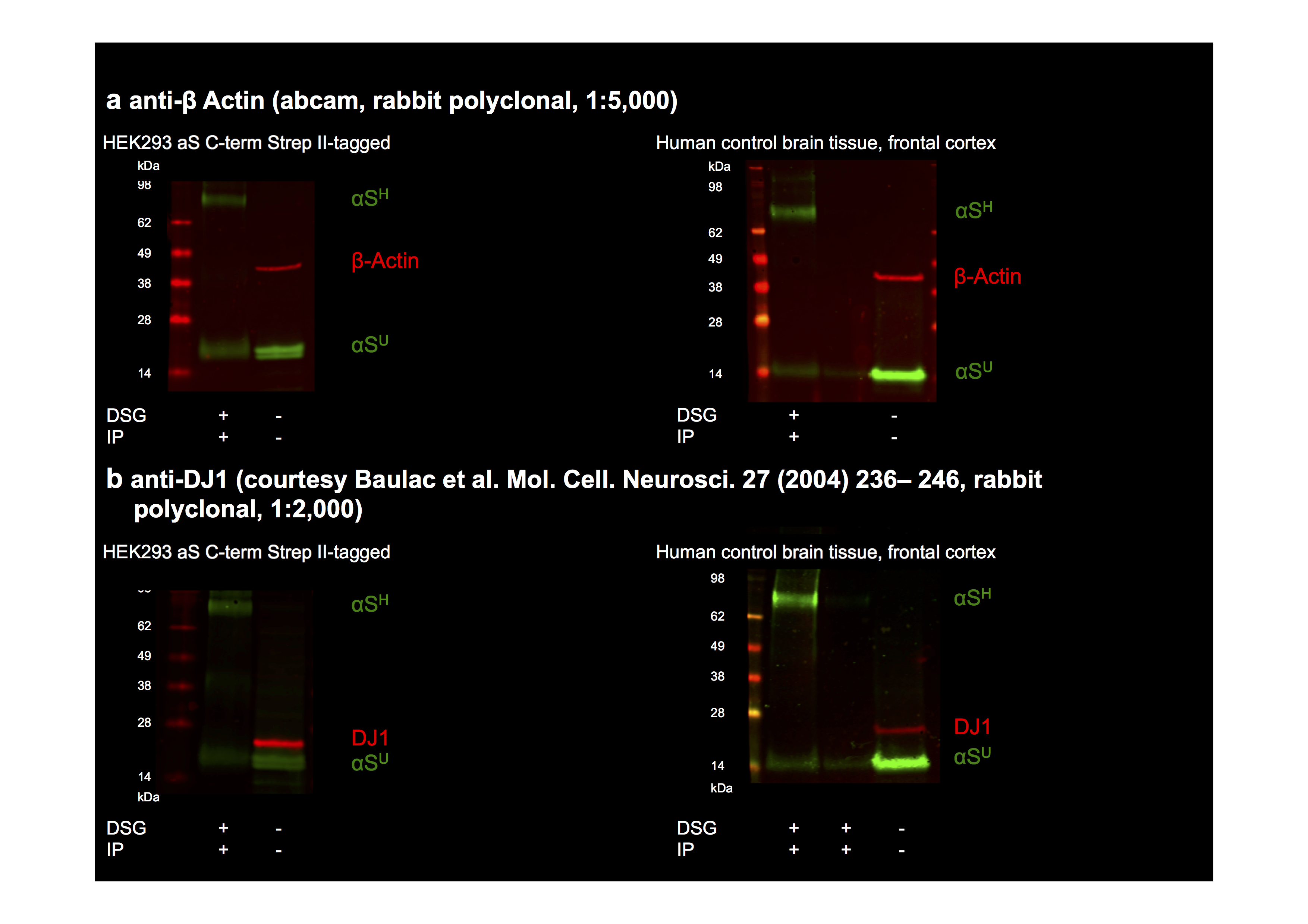


**Fig. 8** Absent co-purification of β-Actin or DJ1 and αS^H^. The crosslinking and immunoprecipitation procedures are described in the Methods section. Samples: HEK cells and human control brain tissue, frontal cortex. HEK293 αS C-term Strep II-tagged lysates were crosslinked and used to purify αS^H^ and αS^U^ using StrepTrap 5ml columns. The αS^H^ and αS^U^ from human brain lysate was immunoprecipitated after crosslinking using the Pierce™ Direct IP Kit. **a** Western blot of immunoprecipitated and crosslinked protein lysate from HEK cells and human brain in comparison to the original lysates demonstrating no co-immunoprecipitation of the β-Actin protein and αS^H^. **b** Western blot of immunoprecipitated and crosslinked protein lysate from HEK cells and human brain in comparison to the original lysates demonstrating no co-immunoprecipitation of the DJ1 protein and αS^H^. The immunoprecipitated human brain sample has been concentrated using an Amicon ultra 50 kDa filter unit and the first lane depicts the concentrated sample, second lane depicts the flow through. DSG “+“ = crosslinked sample. DSG “-“ = non-crosslinked (control) sample. IP “+“ = immunoprecipitated sample, IP “-“ = no immunoprecipitation performed.


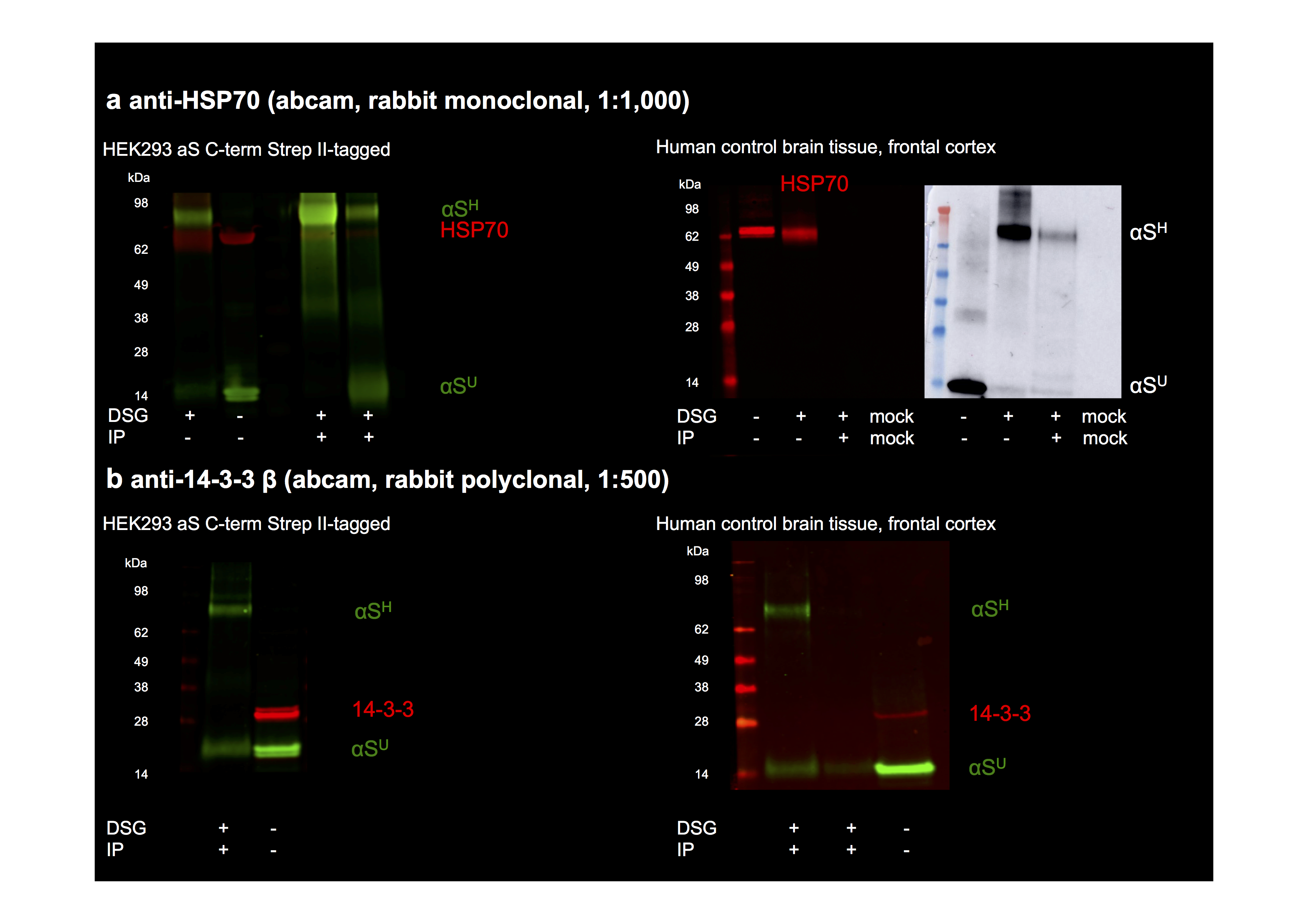
Fig. 9 Absent co-purification of HSP70 or 14-3-3 and αS^H^. The crosslinking and immunoprecipitation procedures are described in the Methods section. Samples: HEK cells and human control brain tissue, frontal cortex. HEK293 αS C-term Strep II-tagged lysates were crosslinked and used to purify αS^H^ and αS^U^ using StrepTrap 5ml columns. The αS^H^ and αS^U^ from human brain lysate was immunoprecipitated after crosslinking using the Pierce™ Direct IP Kit. a Western blot of immunoprecipitated and crosslinked protein lysate from HEK cells and human brain in comparison to the original lysates and one mock IP sample (human brain, no antibody for capturing used). HEK: First lane depicts the crosslinked original lysate, second lane depicts the non-crosslinked original lysate, third and fourth lane depict the αS after crosslinking, immunoprecipitation and SEC to separate αS^H^ and αS^U^. IP human brain samples have been concentrated using Amicon ultra 50 kDa filter units. The Western blots demonstrate no co-immunoprecipitation of HSP70 protein (left) and αS^H^ (right). b Western blot of immunoprecipitated and crosslinked protein lysate from HEK cells and human brain in comparison to the original lysates demonstrating no co-immunoprecipitation of 14-3-3 protein and αS^H^. The immunoprecipitated human brain sample has been concentrated using an Amicon ultra 50 kDa filter unit and the first lane depicts the concentrated sample, second lane depicts the flow through. DSG “+“ = crosslinked sample. DSG “-“ = non-crosslinked (control) sample. IP “+“ = immunoprecipitated sample, IP “-“ = no immunoprecipitation performed.


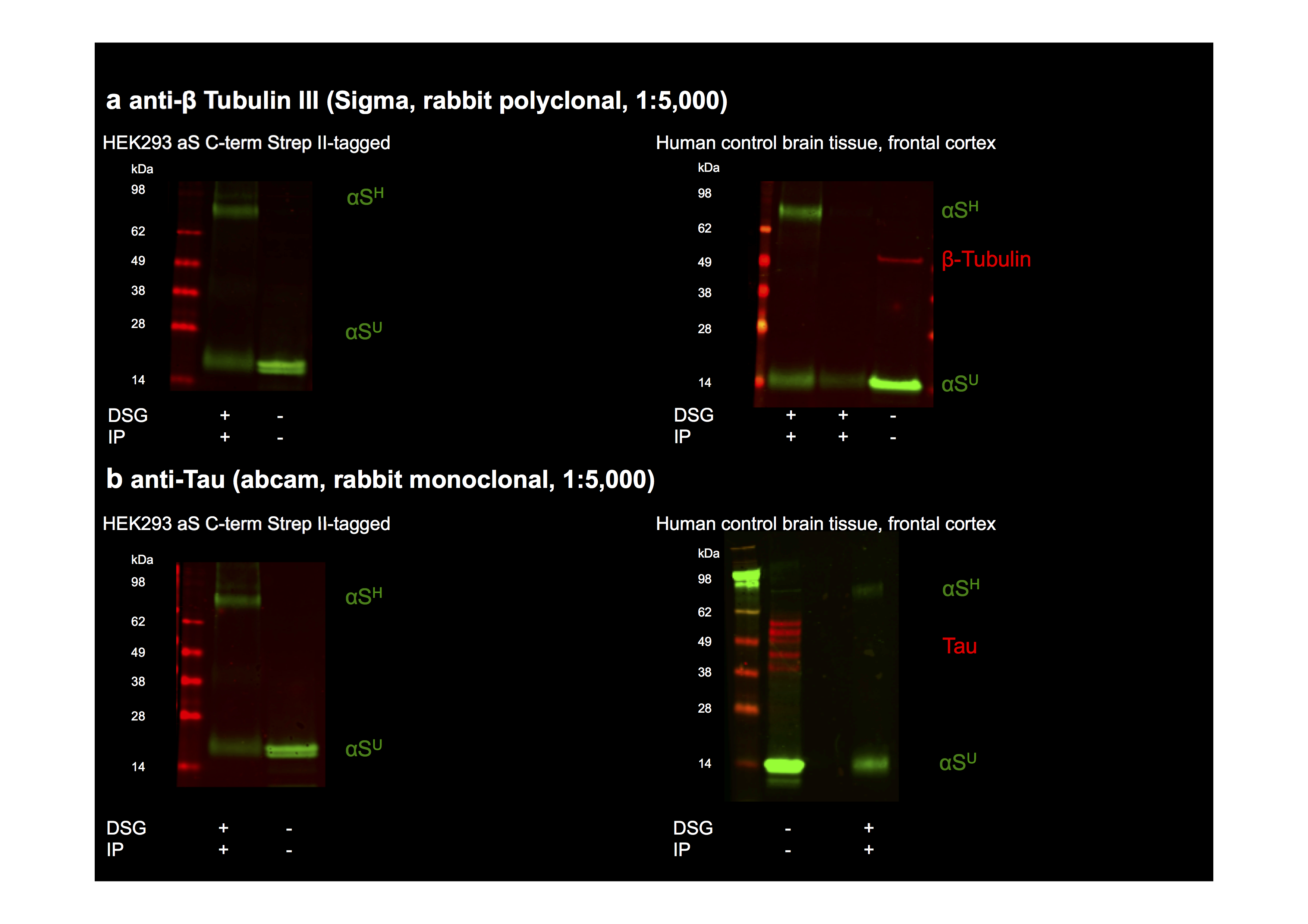


Fig. 10 Absent co-purification of β-Tubulin III or Tau and αS^H^. The crosslinking and immunoprecipitation procedures are described in the Methods section. Samples: HEK cells and human control brain tissue, frontal cortex. HEK293 αS C-term Strep II-tagged lysates were crosslinked and used to purify αS^H^ and αS^U^ using StrepTrap 5ml columns. The αS^H^ and αS^U^ from human brain lysate was immunoprecipitated after crosslinking using the Pierce™ Direct IP Kit. a Western blot of immunoprecipitated and crosslinked protein lysate from HEK cells and human brain in comparison to the original lysates demonstrating no co-immunoprecipitation of β-Tubulin protein and αS^H^ in the brain sample. The immunoprecipitated human brain sample has been concentrated using an Amicon ultra 50 kDa filter unit and the first lane depicts the concentrated sample, second lane depicts the flow through. β Tubulin could not be demonstrated in the original lysate and immunoprecipitated sample of HEK cells. b Western blot of immunoprecipitated and crosslinked protein lysate from HEK cells and human brain in comparison to the original lysates demonstrating no co-immunoprecipitation of tau protein and αS^H^ in the human brain sample. Tau could not be demonstrated in the original lysate and immunoprecipitated sample of HEK cells. DSG “+“ = crosslinked sample. DSG “-“ = non-crosslinked (control) sample. IP “+“ = immunoprecipitated sample, IP “-“ = no immunoprecipitation performed.


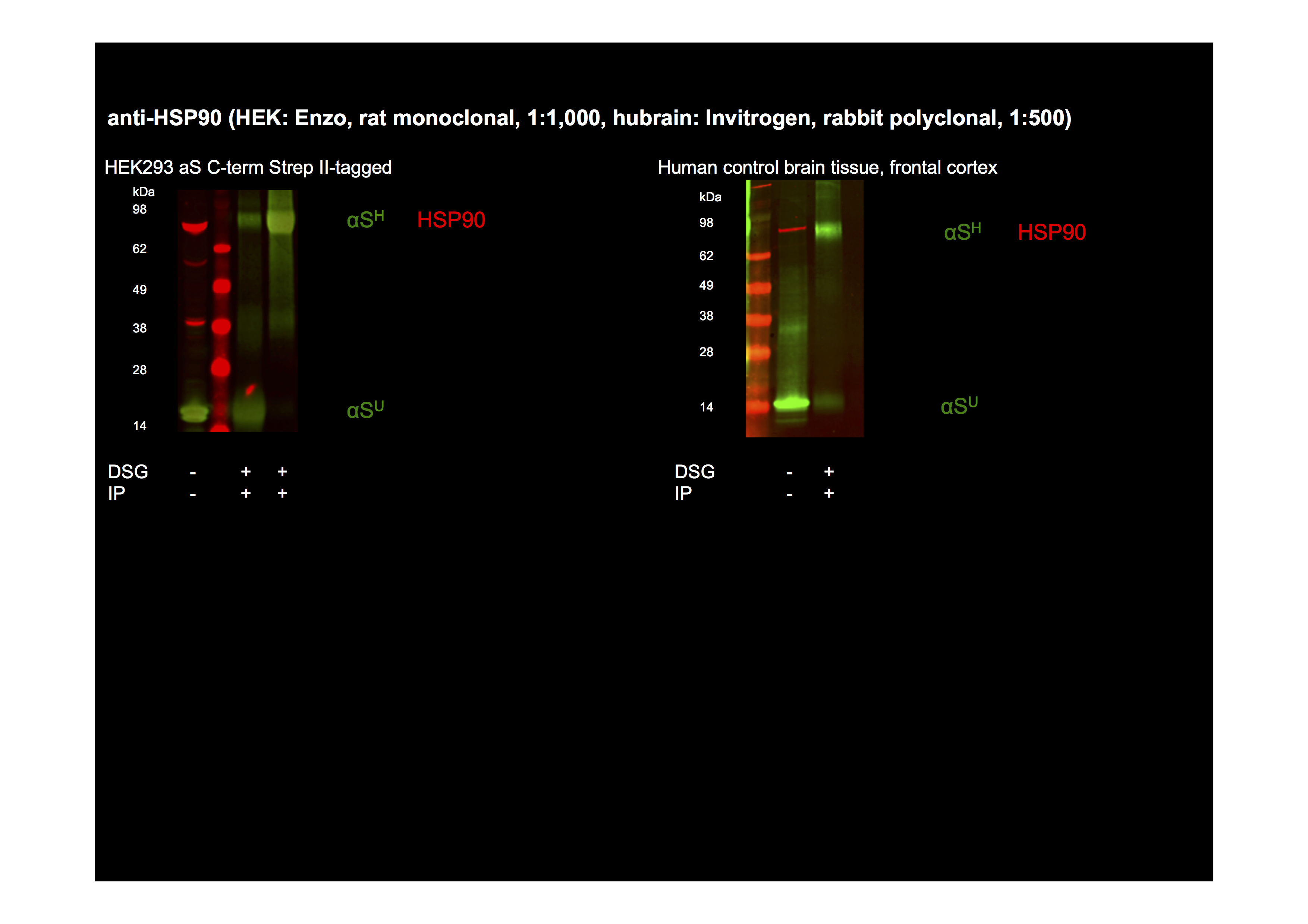
Fig. 11 Absent co-purification of HSP90 and αS^H^. The crosslinking and immunoprecipitation procedures are described in the Methods section. Samples: HEK cells and human control brain tissue, frontal cortex. HEK293 αS C-term Strep II-tagged lysates were crosslinked and used to purify αS^H^ and αS^U^ using StrepTrap 5ml columns. The αS^H^ and αS^U^ from human brain lysate was immunoprecipitated after crosslinking using the Pierce Pierce™ Direct IP Kit. Western blot of immunoprecipitated and crosslinked protein lysate from HEK cells and human brain in comparison to the original lysates demonstrating no co-immunoprecipitation of HSP90 protein and αS^H^. HEK: First lane depicts the non-crosslinked original lysate, second lane and third lane depict the αS after crosslinking, immunoprecipitation and SEC to separate αS^H^ and αS^U^. DSG “+“ = crosslinked sample. DSG “-“ = non-crosslinked (control) sample. IP “+“ = immunoprecipitated sample, IP “-“ = no immunoprecipitation performed.


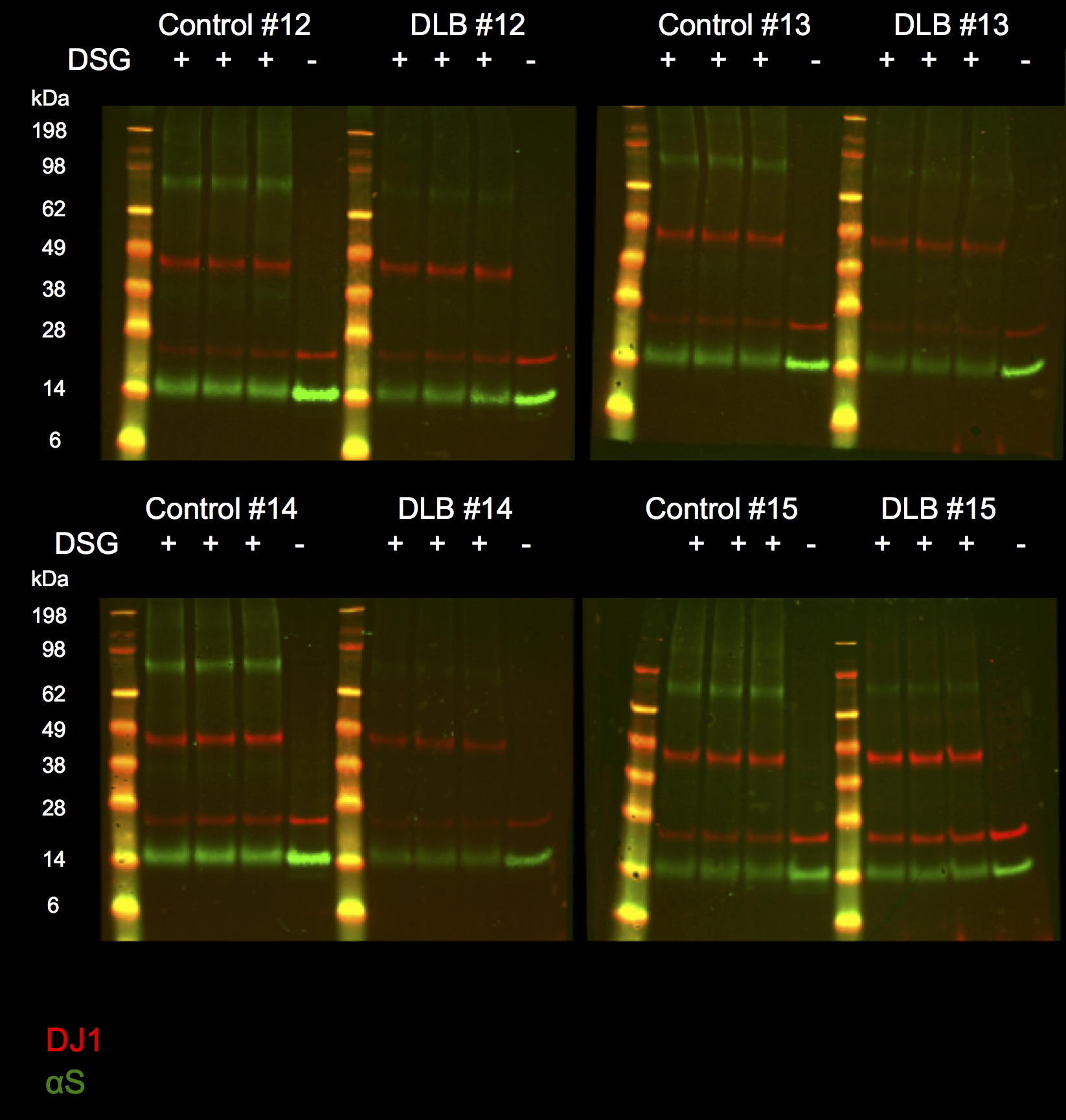


**Fig. 12** Exemplary Western blot of analyzed samples. Western blot of crosslinked frontal cortex (FC) lysate (control vs. DLB, frontal cortex, n=4 each). The crosslinking reaction was performed in technical triplicates alongside with one non-crosslinked (PBS) sample. The Western blot demonstrates reduced αS^H^ / αS^U^ ratios in DLB patients compared to controls. DSG “+“ = crosslinked sample. DSG “-“ = non-crosslinked sample. Green = αS, red = DJ1


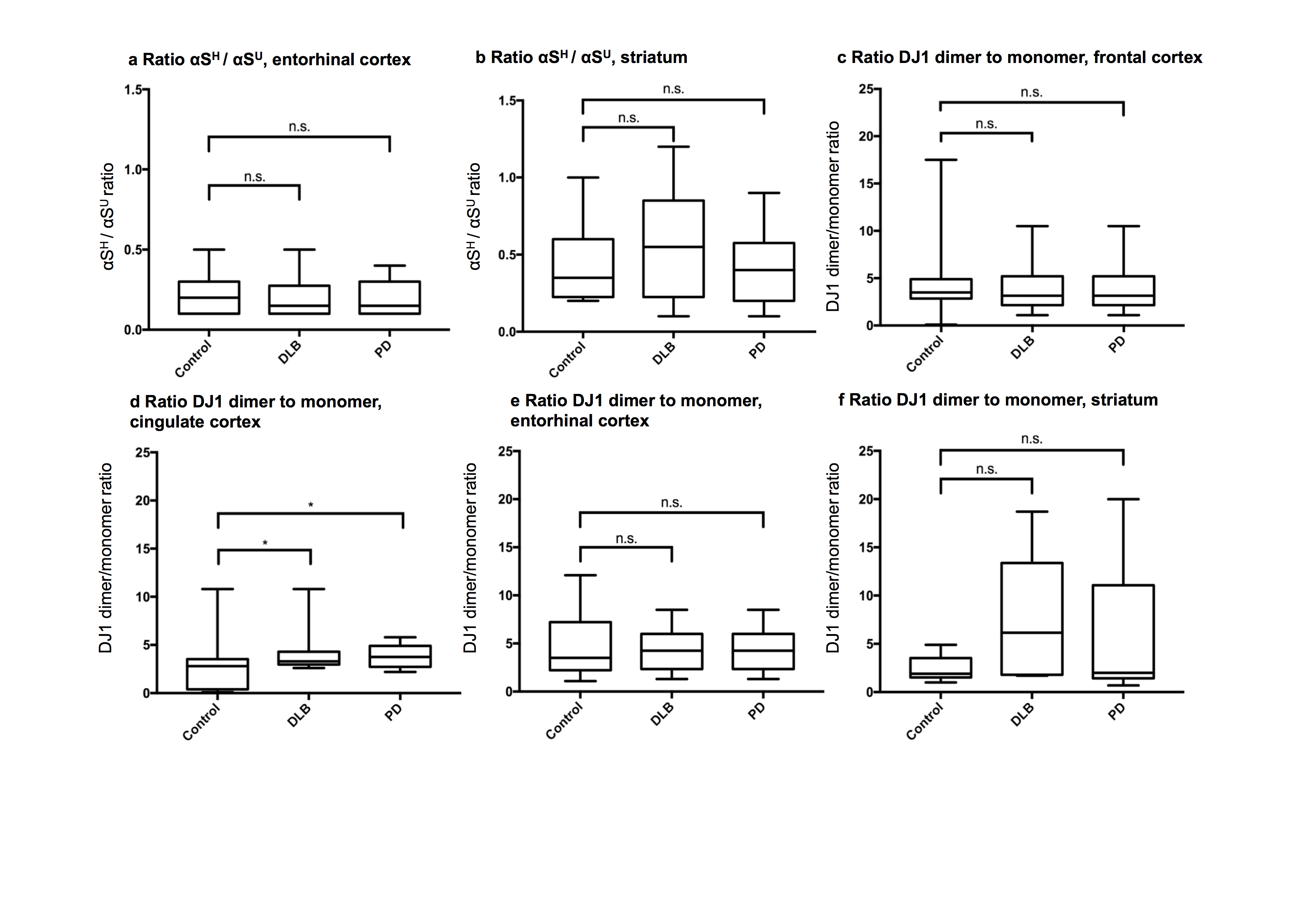


Fig. 13 Similar αS^H^ / αS^U^ and DJ1 dimer to monomer ratios across different brain regions. a No significant alteration of the αS^H^ / αS^U^ equilibria in the entorhinal cortex comparing controls (n = 7) to PD (n = 6) and DLB (n = 6) patients. b No significant alteration of the αS^H^ / αS^U^ equilibria in the striatum comparing controls (n = 6) to PD (n = 6) and DLB (n = 4) patients. The DJ1 serves as an internal control for the crosslinking procedure. Across different brain regions (c frontal cortex e entorhinal cortex, f striatum) no differences in DJ1 dimer to monomer ratios were detected comparing controls, DLB patients and PD patients. d PD and DLB patients exhibited increased DJ1 ratios in the cingulate cortex (p<0.01). Thus, higher αS^H^ / αS^U^ are not due to an overcrosslink of the samples as DLB and PD samples stay low in their αS^H^ / αS^U^ ratios. Entorhinal cortex controls n=7 individuals, DLB n=6 individuals, PD n=6 individuals. Cingulate cortex controls n=7 individuals, DLB n=7 individuals, PD n=6 individuals. Frontal cortex controls n=19 individuals, DLB n=14 individuals, PD n=8 individuals. Striatum controls n=6 individuals, DLB n=4 individuals, PD n=6 individuals. Samples have been analyzed in biological duplicates and technical triplicates. N.s.=not significant.

**
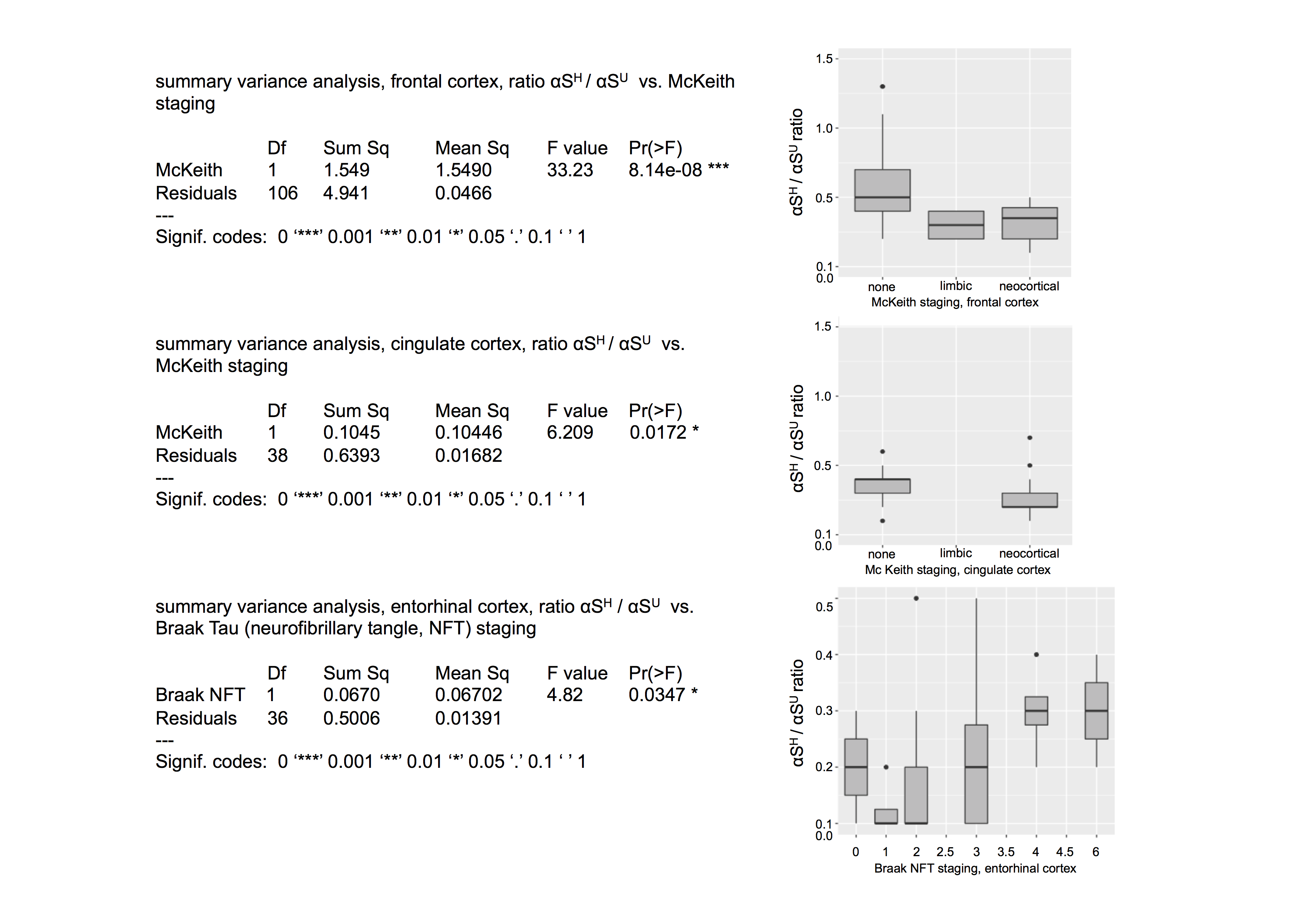
**

**Fig. 14** Significant difference of decreased αS^H^ / αS^U^ ratios and increased McKeith staging (none, limbic, neocortical) of the frontal cortex and cingulate cortex. Significant difference of decreased αS^H^ / αS^U^ ratios and increased Braak neurofibrillary tangle (NFT) staging in the entorhinal cortex.


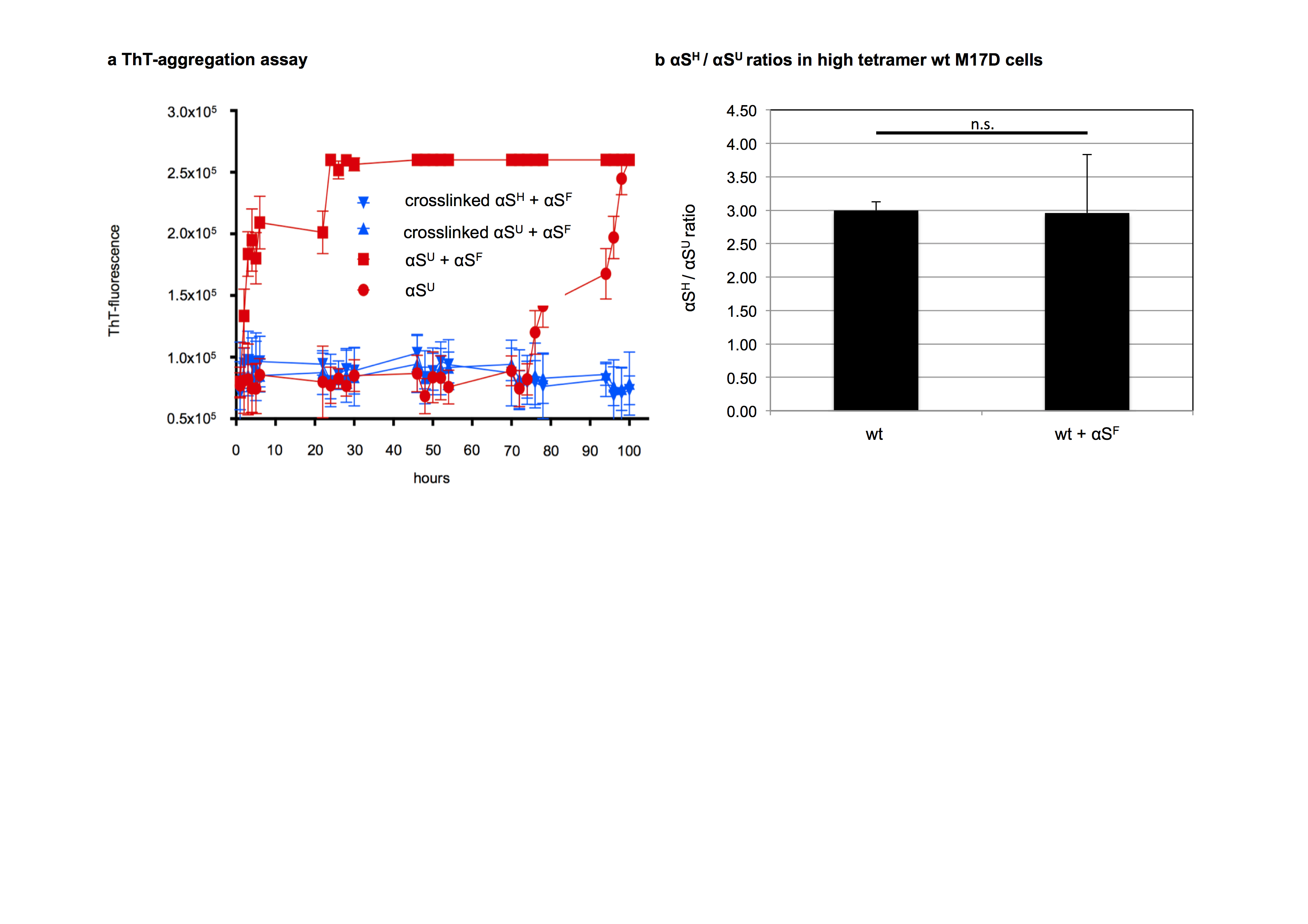


**Fig. 15 a** ThT-aggregation assay of recombinant αS^U^ and crosslinked brain-derived αS^U^ and αS^H^. Comparison between non-crosslinked and crosslinked αS species in a Thioflavin T-aggregation assay. Crosslinked brain-derived αS^U^ and αS^H^ show an absence of insoluble αS formation, with or without seeding of recombinant αS^F^ (αS: αS^F^ 500,000:1). Non-crosslinked recombinant αS^U^ will slowly form ThT-positive fibrils, greatly accelerated by the addition of αS^F^ (αS: αS^F^ 500,000:1) under otherwise identical conditions. **b** αS^F^-seeded M17D high tetramer cell lines maintain αS^H^ / αS^U^ ratios in wt cells, indicating that amyloid aggregation itself does not stimulate αS^H^ destabilization.
